# Cardiovascular effects of auricular stimulation -a systematic review and meta-analysis of randomized controlled clinical trials

**DOI:** 10.3389/fnins.2023.1227858

**Published:** 2023-09-01

**Authors:** Kevin Hua, Mike Cummings, Miriam Bernatik, Benno Brinkhaus, Taras Usichenko, Joanna Dietzel

**Affiliations:** ^1^Institute for Social Medicine, Epidemiology and Health Economics, Berlin Institute of Health, Charité - University Medicine, Corporate Member of Freie Universität Berlin, Humboldt-Universität zu Berlin, Berlin, Germany; ^2^British Medical Acupuncture Society, London, United Kingdom; ^3^International Society for Chinese Medicine, Munich, Germany; ^4^Department for Anesthesiology, University Hospital Greifswald, Greifswald, Germany; ^5^Department of Anesthesia, McMaster University, Hamilton, ON, Canada

**Keywords:** auricular acupuncture, auricular stimulation, cardiovascular, randomized controlled trials, systematic review, transauricular vagus nerve stimulation

## Abstract

**Background:**

The number of randomized controlled trials using auricular stimulation (AS) such as transauricular vagus nerve stimulation, or other auricular electrostimulation or auricular acupuncture or acupressure, in experimental and clinical settings, has increased markedly over the last three decades. This systematic review focusses on cardiovascular effects of auricular stimulation.

**Methods and analysis:**

The following databases were searched: MEDLINE (PubMed), EMBASE, Cochrane Central Register of Controlled Trials (CENTRAL), ISI Web of Science, and Scopus Database. RCTs were reviewed that had been published in English and European languages. Data collection and analysis was conducted by two reviewers independently. Quality and risk assessment of included studies was performed and the meta-analysis of the effect of the most frequently assessed biomarkers.

**Results:**

Altogether, 78 trials were included. 38 studies assessed heart rate (HR), 19 studies analyzed heart rate variability (HRV), 31 studies analyzed blood pressure (BP) and 7 studies were identified that measured oxygen saturation (O2), 2 studies on baroreflex sensitivity and 2 studies on skin conductance were evaluated in this review. 26 studies contained continuous data and were eligible for meta-analysis, 50 trials reported non continuous data and were evaluated descriptively. The overall quality of the studies was moderate to low. AS leads to a significant reduction of HR, the changes though were not considered an adverse reaction. Furthermore, when looking at HRV, AS was able to reduce the LF/HF ratio significantly compared to control procedures. No other cardiovascular parameters (blood pressure, oxygen saturation, baroreflex sensitivity) were changed significantly. AS produced only minor side effects in all trials.

**Conclusion:**

AS can lead to clinically safe reduction of HR and changes in the LF/HF ratio of the HRV, which is presumably via an increase in vagal activity. More research is needed to clarify whether AS can be used to modulate tachycardia or indications with autonomic imbalance.

**Systematic review registration:**

https://www.crd.york.ac.uk/prospero/display_record.php?RecordID=231885 PROSPERO, ID CRD42021231885.

## 1. Introduction

Research evaluating the effectiveness of stimulation of the pinna (auricular stimulation, AS) has markedly increased in the last 30 years. AS trials apply a variety of techniques such as acupuncture or acupressure, or electrostimulation on distinct anatomical regions of the auricle. For an extended list of AS techniques see Table 1. The potential mechanism of AS effects is attributed to the neuroanatomical conditions of the external auricle. It is presumed that AS exerts its effects via the involvement of cranial nerves V, VII and X (Peuker and Filler, 2002). Alderman’s nerve or Arnold’s nerve is a branch of the vagus nerve which forms a receptive field in the pinna of the ear; stimulation in these areas are thought to lead to vagal activation and to the modulation of brain areas involved in stress response, such as the limbic system, locus coeruleus and hypothalamus (Qu et al., 2014; Frangos et al., 2015). Smoking cessation, drug withdrawal, pain relief, heart rhythm disorders, epilepsy, insomnia and depression, and obesity treatments are among the most frequently evaluated conditions (Gates et al., 2006; Lan et al., 2015; Liu et al., 2018; Moura et al., 2019; Mendonça et al., 2020; Yap et al., 2020). Biomarkers, such as blood samples measuring metabolic profiles, inflammatory or immunological markers, anthropometric data such as weight, BMI, as well as cardiological and neurological electrophysiological measurements and functional neuroimaging are used as objective outcomes.

**Table 1:** Examples of auricular stimulation described in clinical trials.

Auricular electric vagal stimulation (AEVS)
Auricular neurostimulation percutaneous electrical nerve field stimulation (PENFS)
Auricular acupuncture
Auricular acupressure
Auricular plaster therapy
Auricular point sticking
Auricular reflexotherapy
Auricular-pressing pill
Auriculomedicine
Daith piercing
Dense cranial electroacupuncture stimulation (DCEAS)
Ear point taping and pressing therapy combined with acupoint-injection
Ear points’ pressing
Ear pressure plaster
Ear-clips
Electrical auricula-vagus-stimulation
He-Ne laser auricular irradiation Intrinsic auricular muscles zone stimulation (IAMZS)
Laser reflexotherapy (only BA?)
Liquid ear acupuncture
Low level transcutaneous vagus nerve stimulation
Low-level tragus stimulation
Low-level tragus stimulation (LLTS)
Low-level transcutaneous electrical vagus nerve stimulation Low level laser therapy (LLLT)
Motor-activated auricular vagus nerve stimulation (MAAVNS) system
Non-vagal auricular stimulation (NVAS)
Otoacupoint pellet pressure
Otopoint-penetrative needling
Photobiomodulation on auriculotherapy points
Photoelectric stimulation of defined ear points
Respiratory-gated auricular vagal afferent nerve stimulation (RAVANS)
Staplepuncture surgical staple implanted in the concha of the ear
Transcutaneous vagus nerve stimulation (tVNS)
Vibrotactile treatment

However, an extensive overview including all types of AS and their effects on cardiovascular biomarkers, is missing. This systematic review was developed following the PRISMA guidelines to explore and evaluate-to our knowledge for the first time—the existing literature regarding the effect on cardiovascular parameters in randomized controlled trials comparing AS with sham AS or AS with no intervention. This review also aimed to investigate whether systemic effects from AS are clinically significant and helps to identify the potential for future clinical research for AS.

### 1.1. Objectives

The aim of this systematic review and meta-analysis is to evaluate the effects of AS on cardiovascular parameters and the safety of AS in healthy individuals and patients.

## 2. Methods

The systematic review protocol has been registered on PROSPERO (ID CRD42020184795). Since all data used in this systematic review have been published, this review does not require ethical approval.

### 2.1. Eligibility criteria for included trials in the review.

#### 2.1.1. Types of trials

The review included only randomized controlled trials (RCTs) in English and other European languages. The funding source was registered. Systematic review and meta-analysis were conducted according to the Cochrane Handbook for Systematic Reviews of Interventions.

#### 2.1.2. Types of participants

The review comprised randomized controlled studies in clinical settings (with patients) and experimental settings with healthy individuals. No restrictions regarding age, gender, or ethnicity of health conditions were made.

#### 2.1.3. Types of interventions

We included all RCTs applying auricular stimulation alone or in addition to routine care. All interventions were eligible from traditional AS (i.e., auricular acupuncture, auricular electroacupuncture, auricular acupressure) to related techniques such as the electrical transcutaneous auricular vagus nerve stimulation (tVNS) in the conchae of the auricle or cranial electrotherapy stimulation (CES) with electrodes clipped to each earlobe. Any comparison with control conditions (sham acupuncture, sham acupressure, placebo stimulation, routine care etc.) were included. We excluded trials that compared one AS to another AS technique.

#### 2.1.4. Types of outcome measures

##### 2.1.4.1. Outcomes

We screened for the following parameters as part of our eligibility review of studies: heart rate (HR), systolic blood pressure (SBP), diastolic blood pressure (DBP), Heart rate variability (HRV,), Low frequency (LF), High frequency (HF), LF/HF ratio und oxygen partial pressure. All cardiovascular biomarkers, that were reported with results were extracted and evaluated, non-continuous data were extracted and evaluated separately. In serial measurements we chose the values at the end of the intervention period. Adverse event reporting was analyzed.

### 2.2. Search methods for identification of trials

#### 2.2.1. Electronic searches.

Two researchers (JD and KH) searched the following databases from inception until 17^th^ November of 2021: MEDLINE (PubMed), EMBASE, Cochrane Central Register of Controlled Trials (CENTRAL), ISI Web of Science, Scopus Database. The search strategy for medline was “randomized controlled trial” OR “controlled clinical trial” OR “randomized” OR “trial” OR “RCT” AND “auricular acupuncture” OR “auricular acupressure” OR “auricular electro-acupuncture” OR “auricular stimulation” OR “auriculotherapy” OR “ear acupuncture” OR “taVNS” OR “auricular vagus nerve stimulation” OR “tVNS” OR “transcutaneous vagus nerve stimulation” OR “transauricular vagus nerve stimulation” OR “percutaneous auricular vagus nerve stimulation” OR “auricular laser stimulation” OR “CES” OR “cranial electrotherapy stimulation.”

### 2.3. Data extraction and management

#### 2.3.1. Trial identification

**Figure 1:**
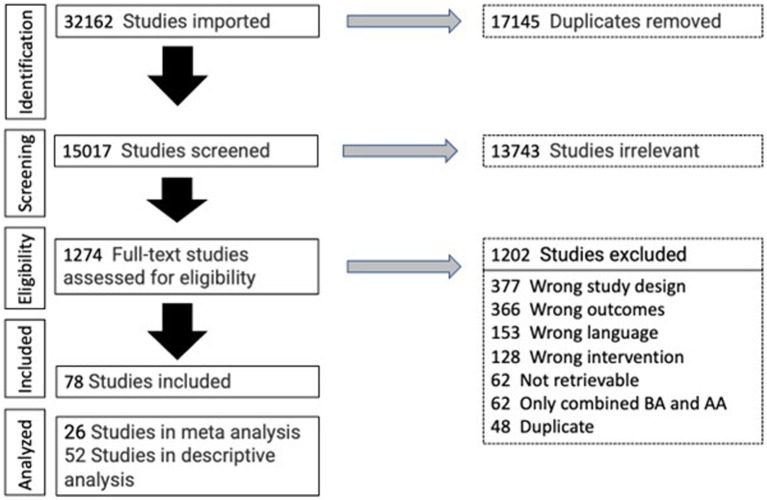
PRISMA flowchart. AA, auricular acupuncture; BA, body acupuncture.

Two researchers (JD, KH) screened independently: titles, abstracts, and full texts for eligibility. Disagreements were resolved by discussion with a third author (TU). If an article did not provide enough information to decide about eligibility, we contacted the trial authors via e-mail. The selection process is depicted in the PRISMA Flow Chart in Figure 1 (Page et al., 2021). Management of selected studies was done with the help of the Covidence software (Covidence systematic review software, Veritas Health Innovation, Melbourne, Australia^1^).

#### 2.3.2. Data extraction and assessment of risk of bias in included trials.

JD and KH independently extracted data and evaluated the methodological quality of each RCT using Risk of Bias domains recommended in the Cochrane Handbook following the RoB 1 tool with some modifications. A consensus procedure was performed before entering the data into Review Manager software (RevMan 5.4. 2020).

#### 2.3.3. Measures of treatment effects and dealing with missing data

For non-continuous outcomes the effect measures of choice were analysed descriptively. Cardiovascular outcomes, that were presented as continuous data were analysed as mean differences with 95% confidence intervals (CI), or as standardized mean differences (SMD). If final means were not reported, we used changes from baseline in the meta-analysis. As well, in case of very different baseline values between groups, changes from baseline were used in the final calculation instead. If relevant numbers of data were missing, we reported it in the Risk of Bias section. We did not apply imputation or other strategies for missing data.

#### 2.3.4. Assessment of heterogeneity

Owing to the broad inclusion criteria a high heterogeneity was to be expected. We only applied random effects meta-analysis (RE), instead of fixed effect meta-analysis (FE) where we considered high heterogeneity a relevant issue.

Heterogeneity was regarded substantial if *T*^2^ is greater than zero and either *I*^2^ is greater than 50% or in case of low *p* value (less than 0.10) in the Chi^2^ test for heterogeneity. The measure T^2^ can be calculated directly from Cochran’s Q. Here, the individual deviations are weighed according to the precision of the respective individual studies, i.e., studies with low random scatter are considered more than studies with lower precision and thus have less influence on the estimation of the meta-estimate. I^2^ according to Higgins/Thompson: *I*^2^ can be interpreted as the ratio of the variance between the studies to the total variance in the meta-analysis.

#### 2.3.5. Assessment of reporting biases

A funnel plot with asymmetry was examined for each of the analyses and is provided in the Supplementary material.

### 2.4. Data synthesis

Fixed-effect meta-analysis were performed initially, in cases of high heterogeneity, random effect analyses were performed. Dichotomous data were analysed separately. The “fixed effects” approach assumes that effects are constant across studies, therefore it is only appropriate when heterogeneity between studies is negligible. In contrast, the “random effects” approach both within-study variance and between-study variance are considered to estimate the aggregate effect. The “random effects” approach is appropriate when heterogeneity between studies is significant and studies are expected to measure different true effects (Borenstein et al., 2010).

A Grading of Recommendations, Assessment, Development and Evaluation (GRADE) was performed for each cardiovascular parameter. With GRADE, the evidence is summarized in a summary-of-findings table, *rating* the certainty of evidence and the relative and absolute treatment effects for each important endpoint.

Subgroup analysis was performed to assess the effects of the different auricular stimulation methods. If the subgroup analysis using FE demonstrated heterogeneity, the data analysis was performed with the RE model. If we detected a wide dispersion of results between studies or limitation of the quality of the results due to methodologically weak studies, a sensitivity analysis was performed excluding outliers or limited to studies with low risk of bias.

## 3. Results

### 3.1. Literature search and analysis

Out of 1.274 trials that were analyzed with full-text analysis a total of 78 trials contained outcomes with cardiovascular parameters.

### 3.2. Data extraction and analysis

The number of trial participants, gender, age, type of intervention and assessment method for cardiovascular parameter are summarized in Table 2 with the overview of the included studies.

**Table 2:** Overview of included studies.

Study ID	Study arms	Type of study	Intervention	Laterality	Length of stimulation (*h*)	Auricular area of stimulation
*Allison et al. (1995)	2	Obesity	Aapres	Unilateral	2016.00	ABVN/GAN
Lu et al. (2012)	2	Psoriasis vulgaris	Aapres	Alternating	1,344.00	ABVN/GAN
*Yeh et al. (2015)	2	Primary hypertension	Aapres + routine	NR	1,680.00	ABVN/GAN
Usichenko et al. (2005)	2	Total hip arthoplasty	Aapunc + routine	Unilateral	96.00	ABVN/GAN
Usichenko et al. (2007)	2	Arthroscopic knee surgery	Aapunc + routine	Unilateral	2400	ABVN/GAN
Hendawy and Abuelnaga, (2020)	2	Hysterectomy	Aapunc + Aapres	NR	3000	ABVN/GAN
la Marca et al. (2010)	4	Experimental	Aapunc	Unilateral	0.50	ABVN
Napadow et al. (2012)	2	Endometriosis	Aapunc	Unilateral	0.50	ABVN/GAN
Sclocco et al. (2019)	2	Experimental	TVNS	Unilateral	NR	ABVN
Wetzel et al. (2011)	2	Total hip arthorplasty	Aapunc	Unilateral	24.00	ABVN/GAN
Usichenko et al. (2006)	2	Total hip arthroplasty	Aapunc	Unilateral	24.00	ABVN/GAN
*Wang et al. (2004)	2	Anesthesia, parental preoperative anxiety	Aapunc	Unilateral	1.00	ABVN/GAN/ATN
Usichenko et al. (2020)	3	Experimental	Aapunc	Bilateral	48.00	ABVN/GAN/ATN
Wang and Kain (2001)	3	Experimental	Aapunc	Bilateral	48.00	ABVN/GAN
Gauthey et al. (2020)	3	Experimental	TVNS	Unilateral	0.17	ABVN
Wu et al. (2020)	2	Ischemic stroke	TVNS + routine	Unilateral	360.00	ABVN
Capone et al. (2017)	2	Chronic stroke	TVNS + routine	Unilateral	240.00	ABVN/GAN/ATN
Yu et al. (2017)	2	STEMI reperfusion	TVNS + routine	Unilateral	2.58	ABVN/GAN/ATN
Hein et al. (2013)	2	Major depression	TVNS + routine	Bilateral	336.00	ABVN
Staley et al. (2020)	2	Hypertension	TVNS	Unilateral	120.00	ABVN
*Gan et al. (2020)	2	Retinopathy of prematurity	Aapres	Bilateral	2.00	ABVN/GAN
*Abdi et al. (2017)	4	Obesity, hypertension	Aapres	Alternating	1,008.00	ABVN/GAN/ATN
*Chen et al. (2017)	2	Heel prick pain at newborns	Aapres	Bilateral	72.00	ABVN/GAN/ATN
Kovacic et al. (2020)	2	Functional abdominal pain disorders	Aapunc	NR	504.00	ABVN/GAN
Luo et al. (2016)	2	Gynecological surgery	Aapres	Bilateral	0.50	ABVN/GAN
*Barker et al. (2006)	2	Hip fracture	Aapres	Bilateral	0.33	ABVN/GAN
*Lin et al. (2011)	2	Experimental	Aapres	Bilateral	1.00	ABVN/GAN/ATN
Ceccherelli et al. (1981)	2	Minor orthopedic or traumatologic surgery	Aapunc	Bilateral	0.75	ABVN/GAN/ATN
Black et al. (2011)	3	Drug addiction	Aapunc	Bilateral	336.00	ABVN/GAN
*Klausenitz et al. (2016)	3	Experimental	Aapunc	bilateral	48.00	ABVN/GAN/ATN
Nakahara et al. (2019)	5	Experimental	Aapunc	Unilateral	0.03	ABVN/GAN
Arai et al. (2013)	2	Hemicolectomy	Aapunc	Bilateral	12.00	ABVN/GAN
*Taylor and Lee (1992)	5	Experimental	CES	Bilateral	0.50	GAN
Wagenseil et al. (2018)	2	Experimental	CES	Bilateral	1.00	GAN
Taylor et al. (2013)	3	Fibromyalgia	CES	Bilateral	1,344.00	GAN
de Couck et al. (2017)	2	Experimental	TVNS	Unilateral	1.00	ABVN/GAN
Johnson et al. (1991)	4	Experimental	TVNS	Unilateral	0.25	ABVN/GAN
*Borges et al. (2021)	3	Experimental	TVNS	Unilateral	0.83	ABVN
Bauer et al. (2016)	2	Epilepsy	TVNS	Unilateral	3,360.00	ABVN/GAN
*Busch et al. (2013)	2	Experimental	TVNS	Unilateral	0.33	ABVN/ATN
*Tobaldini et al. (2019)	2	Experimental	TVNS	Unilateral	0.42	ABVN
*Antonino et al. (2017)	3	Experimental	TVNS	Bilateral	0.25	ABVN/GAN
*Giraudier et al. (2020)	2	Experimental	TVNS	Unilateral	0.38	ABVN
Burger et al. (2017)	2	Experimental	TVNS	Unilateral	NR	ABVN
Capone et al. (2015)	2	Experimental	TVNS	unilateral	1.00	ABVN/GAN/ATN
Keute et al. (2019)	2	Experimental	TVNS	Unilateral	0.50	ABVN
Burger et al. (2016)	2	Experimental	TVNS	Unilateral	0.50	ABVN
Koenig et al. (2021)	2	Major depressive disorder	TVNS	Unilateral	0.50	ABVN
Hasan et al. (2015)	2	Schizophreny	TVNS	Unilateral	2016.00	ABVN
Badran et al. (2018)	2	Experimental	TVNS	Unilateral	1.50	ABVN/GAN
Colzato et al. (2018)	2	Experimental	TVNS	Unilateral	0.67	ABVN/GAN
*Bretherton et al. (2019)	2	Experimental	TVNS	NR	0.25	ABVN/GAN/ATN
*Kuo et al. (2016)	2	Caesarean surgery	Aapres + routine	NR	96.00	ABVN/GAN
Borges et al. (2019)	2	Experimental	TVNS	Unilateral	NR	ABVN
*Sabino-Carvalho et al. (2017)	3	Experimental	TVNS	Bilateral	0.25	ABVN/GAN/ATN
Burger et al. (2018)	2	Experimental	TVNS	Unilateral	0.42	ABVN
Zhu et al. (2021)	2	Functional dyspepsia	TVNS	Bilateral	NR	ABVN
*Clancy et al. (2014)	2	Experimental	TVNS	Unilateral	0.25	ABVN/GAN/ATN
Laqua et al. (2014)	2	Experimental	TVNS	Bilateral	0.50	ABVN
Sellaro et al. (2015)	2	Experimental	TVNS	Unilateral	0.43	ABVN
*Villani et al. (2019)	2	Experimental	TVNS	Unilateral	0.62	ABVN/GAN
*Vosseler et al. (2020)	2	Experimental	TVNS	Unilateral	2.50	ABVN
Stavrakis et al. (2015)	2	Paroxysmal atrial fibrillation	TVNS	Unilateral	1.00	ABVN/GAN/ATN
Jacobs et al. (2015)	2	Experimental	TVNS	Unilateral	0.28	ABVN/GAN
Steenbergen et al. (2015)	2	Experimental	TVNS	Unilateral	0.75	ABVN
*Fischer et al. (2018)	2	Experimental	TVNS	Unilateral	0.60	ABVN
*Ricci et al. (2020)	2	Experimental	TVNS	Unilateral	1.00	ABVN/GAN/ATN
Burger et al. (2019)	2	Experimental	TVNS	Unilateral	0.75	ABVN
*Ventura-Bort et al. (2018)	2	Experimental	TVNS	Unilateral	48.00	ABVN
Borges et al. (2020)	2	Experimental	TVNS	Unilateral	0.27	ABVN
Stavrakis et al. (2020)	2	Paroxysmal atrial fibrillation	TVNS	Unilateral	4,032.00	ABVN/GAN/ATN
Yeo et al. (2014)	3	Obesity	Aapunc + routine	Alternating	NR	ABVN/GAN
Dellovo et al. (2019)	2	Third molar extraction	Aapres	Alternating	120.00	ABVN/GAN/ATN
Strong et al. (2016)	3	Lung cancer	Aapres + routine	Bilateral	48.00	ABVN/GAN
*WANG et al. (2009)	2	OSAS	Aapres	NR	240.00	ABVN/GAN
Karst et al. (2007)	4	Dental surgery	Aapunc	Unilateral	0.42	ABVN/GAN
Széchenyi et al. (2015)	3	Experimental	Aapunc + routine	Bilateral	1	ABVN/GAN
*Killeen et al. (2002)	2	Cocaine addiction	Aapunc	Bilateral	0.75	ABVN/GAN

Studies with (*) were included in meta-analysis. NR, not reported; Aapres, auricular acupressure; Aapunc, auricular acupuncture; tVNS, transauricular vagus nerve stimulation; CES, cranial electrotherapy stimulation; ABVN, auricular branch of vagus nerve; GAN, great auricular nerve; ATN, auriculotemporal nerve.

### 3.3. Quality assessment

**Figure 2:**
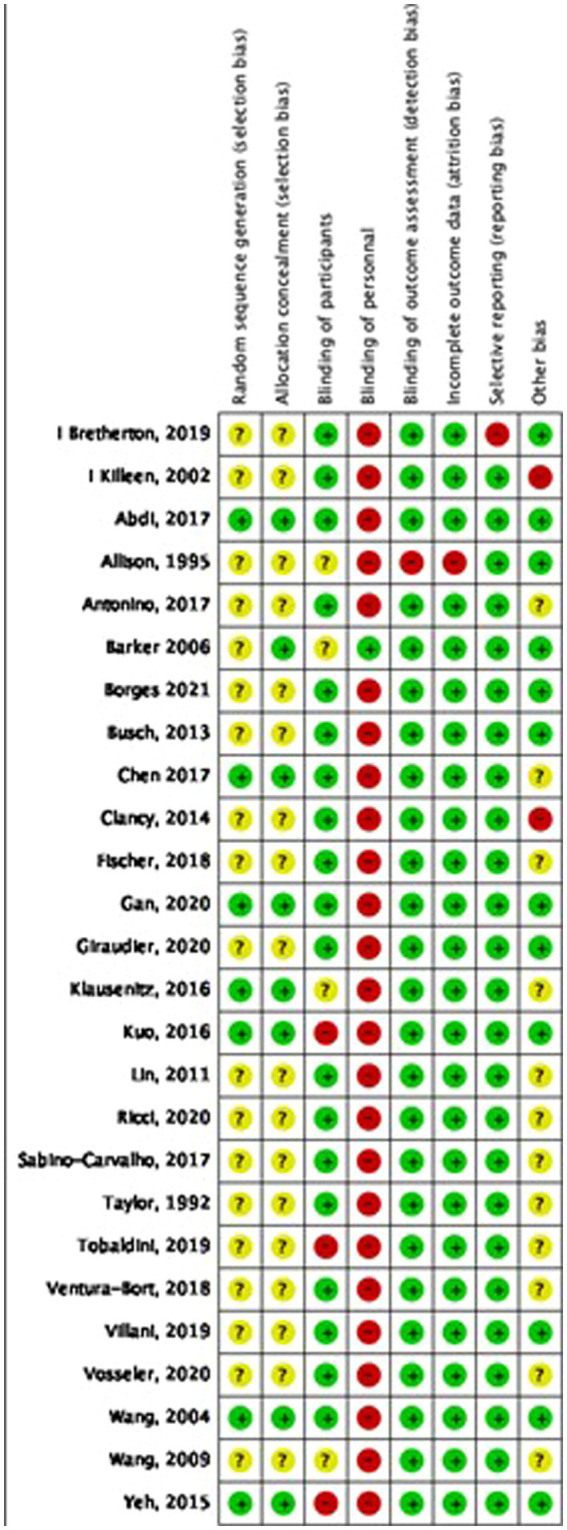
Risk of bias assessment for each study included in the meta-analysis.

**Figure 3:**
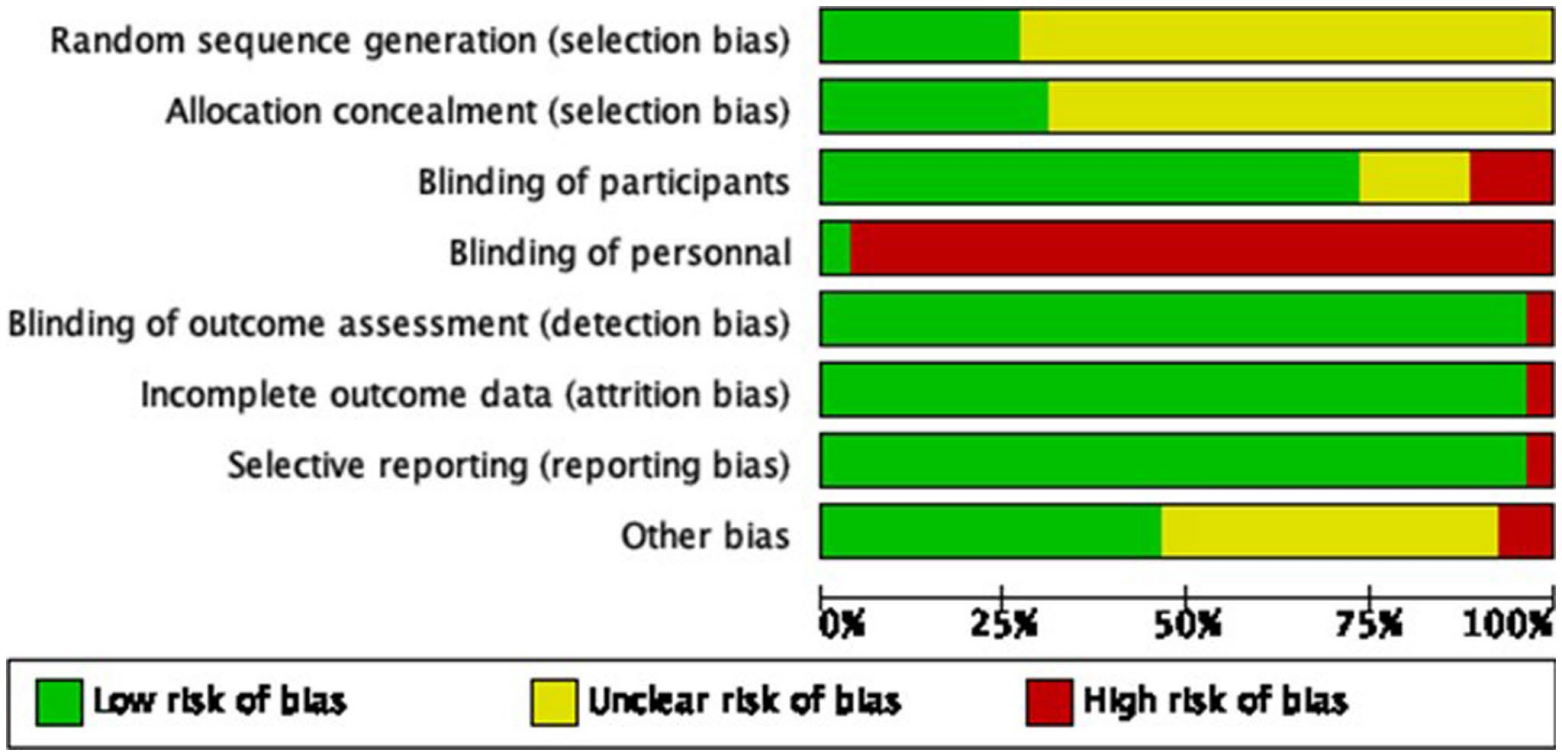
Risk of bias graph across all included studies.

The analysis of study quality was performed for the included studies in the meta-analysis. The overall study quality was moderate (see Figures 2, 3). Missing data did not bias the review findings in general.

### 3.4. Baseline characteristics

In total 3,777 patients were included in the systematic review of cardiovascular parameters. 55,5% were female. Four studies reported no information of the sex of the included population (Taylor and Lee, 1992; Strong et al., 2016; Abdi et al., 2017; Dellovo et al., 2019). The age of the patients ranged between 4 days and 86 years, with a median of 30 years. One study did not provide information about age (Strong et al., 2016).

### 3.5. Comparison of trial designs

We identified several trial designs. Sixty studies used a two-arm design (Ceccherelli et al., 1981; Allison et al., 1995; Killeen et al., 2002; Wang et al., 2004, 2009; Usichenko et al., 2005, 2006, 2007; Barker et al., 2006; Lin et al., 2011; Wetzel et al., 2011; Lu et al., 2012; Napadow et al., 2012; Arai et al., 2013; Busch et al., 2013; Hein et al., 2013; Clancy et al., 2014; Laqua et al., 2014; Capone et al., 2015, 2017; Hasan et al., 2015; Jacobs et al., 2015; Sellaro et al., 2015; Stavrakis et al., 2015, 2020; Steenbergen et al., 2015; Yeh et al., 2015; Bauer et al., 2016; Burger et al., 2016, 2017, 2018, 2019; Kuo et al., 2016; Luo et al., 2016; Chen et al., 2017; de Couck et al., 2017; Yu et al., 2017; Badran et al., 2018; Colzato et al., 2018; Fischer et al., 2018; Ventura-Bort et al., 2018; Wagenseil et al., 2018; Borges et al., 2019, 2020; Bretherton et al., 2019; Dellovo et al., 2019; Keute et al., 2019; Sclocco et al., 2019; Tobaldini et al., 2019; Villani et al., 2019; Gan et al., 2020; Giraudier et al., 2020; Hendawy and Abuelnaga, 2020; Kovacic et al., 2020; Ricci et al., 2020; Staley et al., 2020; Vosseler et al., 2020; Wu et al., 2020; Koenig et al., 2021; Zhu et al., 2021), 12 studies had three arms (Wang and Kain, 2001; Black et al., 2011; Taylor et al., 2013; Yeo et al., 2014; Széchenyi et al., 2015; Klausenitz et al., 2016; Strong et al., 2016; Antonino et al., 2017; Sabino-Carvalho et al., 2017; Gauthey et al., 2020; Usichenko et al., 2020; Borges et al., 2021) and 4 studies performed a four arm trial (Johnson et al., 1991; Karst et al., 2007; la Marca et al., 2010; Abdi et al., 2017), and 2 studies compared five arms (Taylor and Lee, 1992; Nakahara et al., 2019). 40 studies were conducted in healthy volunteers in an experimental setting (Johnson et al., 1991; Taylor and Lee, 1992; Wang and Kain, 2001; la Marca et al., 2010; Lin et al., 2011; Busch et al., 2013; Clancy et al., 2014; Laqua et al., 2014; Capone et al., 2015; Jacobs et al., 2015; Sellaro et al., 2015; Steenbergen et al., 2015; Széchenyi et al., 2015; Burger et al., 2016, 2017, 2018, 2019; Klausenitz et al., 2016; Antonino et al., 2017; de Couck et al., 2017; Sabino-Carvalho et al., 2017; Badran et al., 2018; Colzato et al., 2018; Fischer et al., 2018; Ventura-Bort et al., 2018; Wagenseil et al., 2018; Borges et al., 2019, 2020, 2021; Bretherton et al., 2019; Keute et al., 2019; Nakahara et al., 2019; Sclocco et al., 2019; Tobaldini et al., 2019; Villani et al., 2019; Gauthey et al., 2020; Giraudier et al., 2020; Ricci et al., 2020; Usichenko et al., 2020; Vosseler et al., 2020), while 38 evaluated AS in patients in a clinical setting (Ceccherelli et al., 1981; Allison et al., 1995; Killeen et al., 2002; Wang et al., 2004, 2009; Usichenko et al., 2005, 2006, 2007; Barker et al., 2006; Karst et al., 2007; Black et al., 2011; Wetzel et al., 2011; Lu et al., 2012; Napadow et al., 2012; Arai et al., 2013; Hein et al., 2013; Taylor et al., 2013; Yeo et al., 2014; Hasan et al., 2015; Stavrakis et al., 2015, 2020; Yeh et al., 2015; Bauer et al., 2016; Kuo et al., 2016; Luo et al., 2016; Strong et al., 2016; Abdi et al., 2017; Capone et al., 2017; Chen et al., 2017; Yu et al., 2017; Dellovo et al., 2019; Gan et al., 2020; Hendawy and Abuelnaga, 2020; Kovacic et al., 2020; Staley et al., 2020; Wu et al., 2020; Koenig et al., 2021; Zhu et al., 2021).

We identified different control groups. Most studies (62 in total) were sham controlled trials (Johnson et al., 1991; Taylor and Lee, 1992; Wang and Kain, 2001; Wang et al., 2004; Usichenko et al., 2005, 2006, 2007; Barker et al., 2006; Karst et al., 2007; la Marca et al., 2010; Black et al., 2011; Lin et al., 2011; Wetzel et al., 2011; Napadow et al., 2012; Busch et al., 2013; Hein et al., 2013; Taylor et al., 2013; Clancy et al., 2014; Laqua et al., 2014; Yeo et al., 2014; Capone et al., 2015, 2017; Hasan et al., 2015; Jacobs et al., 2015; Sellaro et al., 2015; Stavrakis et al., 2015, 2020; Steenbergen et al., 2015; Burger et al., 2016, 2017, 2018, 2019; Klausenitz et al., 2016; Luo et al., 2016; Strong et al., 2016; Abdi et al., 2017; Antonino et al., 2017; Chen et al., 2017; de Couck et al., 2017; Sabino-Carvalho et al., 2017; Badran et al., 2018; Colzato et al., 2018; Fischer et al., 2018; Ventura-Bort et al., 2018; Wagenseil et al., 2018; Borges et al., 2019, 2020, 2021; Bretherton et al., 2019; Keute et al., 2019; Sclocco et al., 2019; Villani et al., 2019; Gan et al., 2020; Gauthey et al., 2020; Giraudier et al., 2020; Kovacic et al., 2020; Ricci et al., 2020; Staley et al., 2020; Vosseler et al., 2020; Wu et al., 2020; Koenig et al., 2021). Sham interventions were electrodes without currents, or tVNS at parts of the auricle without vagal innervation, acupuncture or acupressure at selected points with another function, or empty acupressure without the pressing bead. Other trials had active controls (Taylor and Lee, 1992; Wang and Kain, 2001; la Marca et al., 2010; Yeo et al., 2014; Bauer et al., 2016; Klausenitz et al., 2016; Badran et al., 2018; Hendawy and Abuelnaga, 2020; Usichenko et al., 2020), no intervention (Ceccherelli et al., 1981; Johnson et al., 1991; Taylor and Lee, 1992; la Marca et al., 2010; Arai et al., 2013; Nakahara et al., 2019; Tobaldini et al., 2019), or routine care control (Ceccherelli et al., 1981; Karst et al., 2007; Lu et al., 2012; Yeh et al., 2015; Kuo et al., 2016; Strong et al., 2016; Dellovo et al., 2019).

### 3.6. Study interventions

Forty-five studies used noninvasive electrostimulation devices, among them 42 tVNS (Johnson et al., 1991; Busch et al., 2013; Hein et al., 2013; Clancy et al., 2014; Laqua et al., 2014; Capone et al., 2015, 2017; Hasan et al., 2015; Jacobs et al., 2015; Sellaro et al., 2015; Stavrakis et al., 2015, 2020; Steenbergen et al., 2015; Bauer et al., 2016; Burger et al., 2016, 2017, 2018, 2019; Antonino et al., 2017; de Couck et al., 2017; Sabino-Carvalho et al., 2017; Yu et al., 2017; Badran et al., 2018; Colzato et al., 2018; Fischer et al., 2018; Ventura-Bort et al., 2018; Borges et al., 2019, 2020, 2021; Bretherton et al., 2019; Keute et al., 2019; Sclocco et al., 2019; Tobaldini et al., 2019; Villani et al., 2019; Gauthey et al., 2020; Giraudier et al., 2020; Ricci et al., 2020; Staley et al., 2020; Vosseler et al., 2020; Wu et al., 2020; Koenig et al., 2021; Zhu et al., 2021) and 3 studies used CES (Taylor and Lee, 1992; Taylor et al., 2013; Wagenseil et al., 2018). 14 studies used auricular acupressure (Allison et al., 1995; Barker et al., 2006; WANG et al., 2009; Lin et al., 2011; Lu et al., 2012; Yeh et al., 2015; Kuo et al., 2016; Luo et al., 2016; Strong et al., 2016; Abdi et al., 2017; Chen et al., 2017; Dellovo et al., 2019; Gan et al., 2020; Hendawy and Abuelnaga, 2020) and 20 studies investigated auricular acupuncture (Ceccherelli et al., 1981; Wang and Kain, 2001; Killeen et al., 2002; Wang et al., 2004; Usichenko et al., 2005, 2006, 2007, 2020; Karst et al., 2007; la Marca et al., 2010; Black et al., 2011; Wetzel et al., 2011; Napadow et al., 2012; Arai et al., 2013; Yeo et al., 2014; Széchenyi et al., 2015; Klausenitz et al., 2016; Nakahara et al., 2019; Hendawy and Abuelnaga, 2020; Kovacic et al., 2020). Three research groups used additional electrostimulation with the auricular acupuncture (la Marca et al., 2010; Napadow et al., 2012; Kovacic et al., 2020). In terms of methodology, the included studies also varied in the choice of stimulation modality. 46 study groups performed unilateral stimulation of the ear. 20 study groups performed stimulation on both ears (Ceccherelli et al., 1981; Taylor and Lee, 1992; Wang and Kain, 2001; Barker et al., 2006; Black et al., 2011; Lin et al., 2011; Arai et al., 2013; Hein et al., 2013; Taylor et al., 2013; Klausenitz et al., 2016; Luo et al., 2016; Antonino et al., 2017; Chen et al., 2017; Sabino-Carvalho et al., 2017; Wagenseil et al., 2018; Gan et al., 2020; Usichenko et al., 2020; Zhu et al., 2021)la (Laqua et al., 2014; Strong et al., 2016). 4 studies performed alternating stimulation of both ears (Lu et al., 2012; Yeo et al., 2014; Abdi et al., 2017; Dellovo et al., 2019). 6 studies did not provide information on stimulation (WANG et al., 2009; Yeh et al., 2015; Kuo et al., 2016; Bretherton et al., 2019; Hendawy and Abuelnaga, 2020; Kovacic et al., 2020).

The 48 studies that conducted electrical stimulation provided information on the used frequency (Johnson et al., 1991; Taylor and Lee, 1992; la Marca et al., 2010; Napadow et al., 2012; Busch et al., 2013; Hein et al., 2013; Taylor et al., 2013; Clancy et al., 2014; Laqua et al., 2014; Capone et al., 2015, 2017; Hasan et al., 2015; Jacobs et al., 2015; Sellaro et al., 2015; Stavrakis et al., 2015, 2020; Steenbergen et al., 2015; Bauer et al., 2016; Burger et al., 2016, 2017, 2018, 2019; Antonino et al., 2017; de Couck et al., 2017; Sabino-Carvalho et al., 2017; Yu et al., 2017; Badran et al., 2018; Colzato et al., 2018; Fischer et al., 2018; Ventura-Bort et al., 2018; Wagenseil et al., 2018; Borges et al., 2019, 2020, 2021; Bretherton et al., 2019; Keute et al., 2019; Sclocco et al., 2019; Tobaldini et al., 2019; Villani et al., 2019; Gauthey et al., 2020; Giraudier et al., 2020; Hendawy and Abuelnaga, 2020; Ricci et al., 2020; Staley et al., 2020; Vosseler et al., 2020; Wu et al., 2020; Koenig et al., 2021; Zhu et al., 2021). Median frequency was 25 Hz ranging between 0,5 Hz and 100 Hz. In 36 trials that presented information on the current (Taylor and Lee, 1992; Napadow et al., 2012; Busch et al., 2013; Hein et al., 2013; Taylor et al., 2013; Clancy et al., 2014; Capone et al., 2015; Jacobs et al., 2015; Sellaro et al., 2015; Steenbergen et al., 2015; Bauer et al., 2016; Burger et al., 2016, 2017, 2018, 2019; Antonino et al., 2017; de Couck et al., 2017; Sabino-Carvalho et al., 2017; Colzato et al., 2018; Fischer et al., 2018; Ventura-Bort et al., 2018; Wagenseil et al., 2018; Borges et al., 2019, 2020, 2021; Bretherton et al., 2019; Keute et al., 2019; Sclocco et al., 2019; Tobaldini et al., 2019; Gauthey et al., 2020; Giraudier et al., 2020; Ricci et al., 2020; Stavrakis et al., 2020; Vosseler et al., 2020; Koenig et al., 2021; Zhu et al., 2021), the median was 1,15 mA, with a range between 0,1 mA and 45 mA. Concerning pulse width 41 studies reported information (la Marca et al., 2010; Napadow et al., 2012; Busch et al., 2013; Clancy et al., 2014; Laqua et al., 2014; Capone et al., 2015, 2017; Hasan et al., 2015; Jacobs et al., 2015; Sellaro et al., 2015; Stavrakis et al., 2015, 2020; Steenbergen et al., 2015; Bauer et al., 2016; Burger et al., 2016, 2017, 2018, 2019; Antonino et al., 2017; de Couck et al., 2017; Sabino-Carvalho et al., 2017; Yu et al., 2017; Badran et al., 2018; Colzato et al., 2018; Fischer et al., 2018; Ventura-Bort et al., 2018; Borges et al., 2019, 2020, 2021; Bretherton et al., 2019; Keute et al., 2019; Sclocco et al., 2019; Tobaldini et al., 2019; Villani et al., 2019; Gauthey et al., 2020; Giraudier et al., 2020; Ricci et al., 2020; Staley et al., 2020; Wu et al., 2020; Koenig et al., 2021; Zhu et al., 2021), the median pulse width was 250 μs ranging between 200 μs and 500 μs. Regarding the duty cycle 25 studies provided information (Johnson et al., 1991; Napadow et al., 2012; Capone et al., 2015, 2017; Hasan et al., 2015; Sellaro et al., 2015; Steenbergen et al., 2015; Bauer et al., 2016; Burger et al., 2016, 2018, 2019; de Couck et al., 2017; Yu et al., 2017; Colzato et al., 2018; Fischer et al., 2018; Ventura-Bort et al., 2018; Borges et al., 2019, 2020; Keute et al., 2019; Sclocco et al., 2019; Giraudier et al., 2020; Ricci et al., 2020; Vosseler et al., 2020; Wu et al., 2020; Koenig et al., 2021; Zhu et al., 2021), the median duty cycle was 30s with a range of 0,5 s to 300 s. Two trials performed continuous stimulation (Bretherton et al., 2019; Borges et al., 2021). In contrast only 2 studies stated information about the used voltage with 265 mV (la Marca et al., 2010) and 800 mV (Hendawy and Abuelnaga, 2020). For details of stimulation parameters and anatomic region of the pinna see Supplementary Table S2

The mean duration of stimulation was 285 h with a range from 20 min to 168 days. 5 trials did not provide information about stimulation duration (Yeo et al., 2014; Burger et al., 2017; Borges et al., 2019; Sclocco et al., 2019; Zhu et al., 2021). Short-term stimulations were usually performed with wither an electrical device such as the tVNS or mechanical stimulation such as auricular acupressure or acupuncture. The longest stimulation was performed with the Parasym stimulation device. The included population was scheduled to receive stimulation daily for 1 h over 6 months (Stavrakis et al., 2020).

In terms of selection of the auricular area of stimulation, the acupuncture and acupressure studies applied stimulation to single acupuncture points, such as the auricular points “Shenmen” (MA-TF 1) or “Lung” (MA-IC1). For the most frequently used ear- acupuncture points see Supplementary Figure S1.

**Figure 4:**
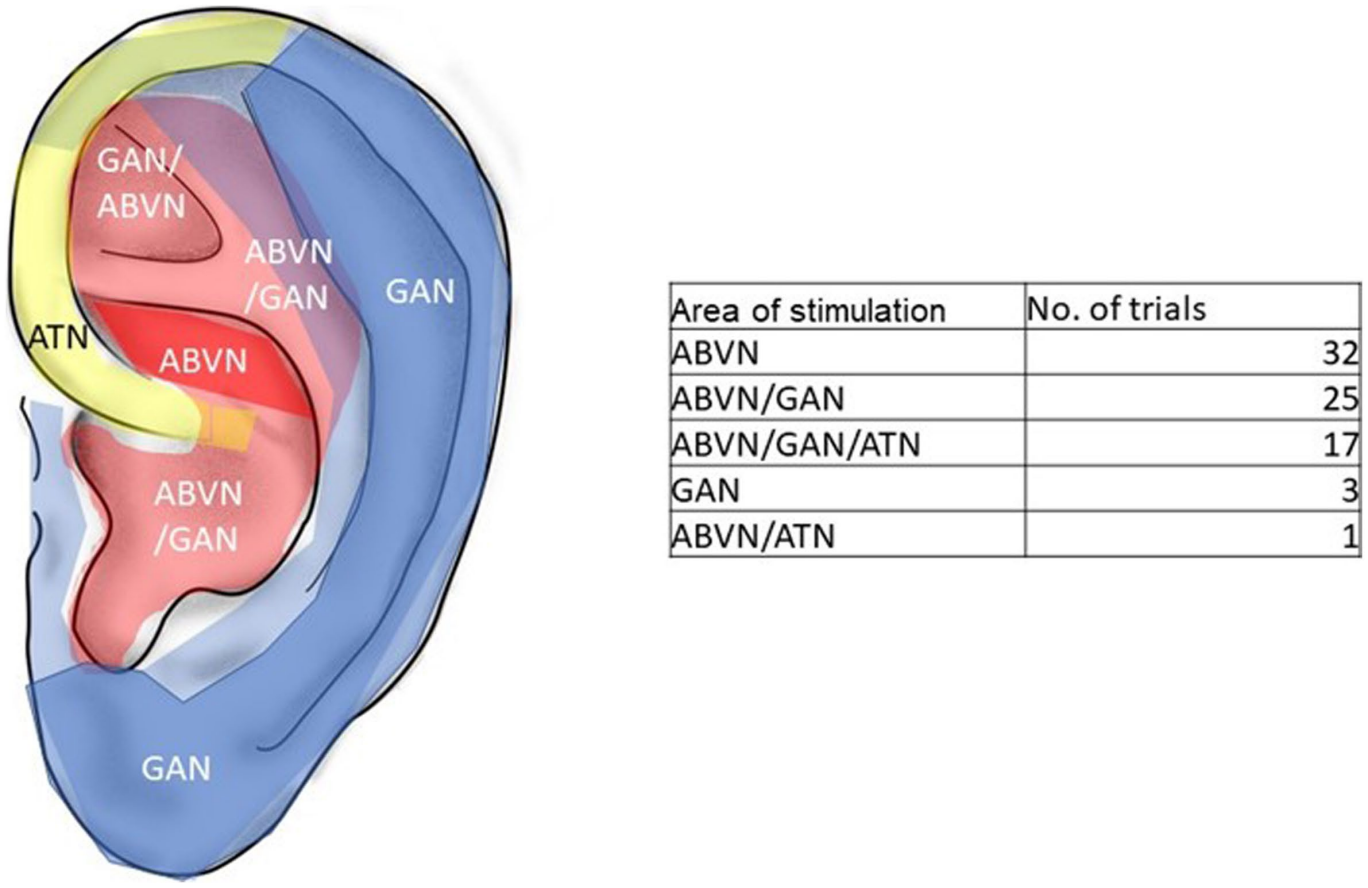
Innervation of the pinna and area of stimulation in the trials. Red: ABVN-auricle branch of the vagus nerve, blue: GAN-great auricular nerve. Yellow: ATN-auriculo-temporal nerve. Modified from Peuker and Filler (2002).

Forty-seven studies used tVNS or CES as the stimulation variant. Interventions such as tVNS or CES apply electrical current to broader areas of the auricle such as the cymba concha or the tragus in tVNS or the earlobes such as in CES. According to Peuker and Filler (2002), the cymba conchae is innervated by the auricular branch of the nervus vagus (ABNV), the tragus has a mixed innervation of ABNV plus great auricular nerve (GAN, a superficial branch of the cervical plexus) and the earlobe is innervated primarily by the GAN and is therefore frequently used as sham- control. For the most frequently used areas of stimulation in the 78 included trials see Figure 4.

### 3.7. Outcomes and safety parameters

Thirty-eight studies assessed heart rate (HR), 19 studies analyzed heart rate variability (HRV), 31 studies analyzed blood pressure (BP) and 7 studies were identified that measured oxygen saturation. In addition, 2 studies on baroreflex and 2 studies skin conductance were evaluated in this review.

Of the 76 studies identified, 26 studies contained continuous data and were eligible for meta-analysis, 50 trials reported non continuous data and were evaluated descriptively (see Table 3).

**Table 3:** Descriptive analysis of studies with non-continuous data.

Outcome and no. of RCTs	Only in AS group	No difference between AS and controls
Heart rate *N* = 25	Reduction: 3 (Ceccherelli et al., 1981; Steenbergen et al., 2015; Luo et al., 2016)	22 (Johnson et al., 1991; Wang and Kain, 2001; Usichenko et al., 2005, 2006, 2007, 2020; Black et al., 2011; Wetzel et al., 2011; Hein et al., 2013; Capone et al., 2015, 2017; Sellaro et al., 2015; Badran et al., 2018; Burger et al., 2018; Wagenseil et al., 2018; Nakahara et al., 2019; Villani et al., 2019; Borges et al., 2020; Hendawy and Abuelnaga, 2020; Kovacic et al., 2020; Wu et al., 2020; Koenig et al., 2021)
Blood pressure *N* = 22	Reduction: 4 (Ceccherelli et al., 1981; Luo et al., 2016; Hendawy and Abuelnaga, 2020; Usichenko et al., 2020)	19 (Johnson et al., 1991; Wang and Kain, 2001; Usichenko et al., 2005, 2006, 2007, 2020; Black et al., 2011; Wetzel et al., 2011; Taylor et al., 2013; Laqua et al., 2014; Yeo et al., 2014; Capone et al., 2015, 2017; Sellaro et al., 2015; Villani et al., 2019; Gauthey et al., 2020; Kovacic et al., 2020; Staley et al., 2020; Wu et al., 2020)
Heart rate variability *N* = 10	Increase: 2 (Arai et al., 2013; Bretherton et al., 2019)	8 (Napadow et al., 2012; Burger et al., 2016, 2018, 2019; de Couck et al., 2017; Borges et al., 2019; Gauthey et al., 2020; Koenig et al., 2021)
High frequency power *N* = 4	Increase: 2 (Sclocco et al., 2019; Zhu et al., 2021) Reduction: 1 (Stavrakis et al., 2020)	1 (Laqua et al., 2014)
Low frequency power *N* = 2	Increase: 1 (Stavrakis et al., 2020) Reduction: 1 (Zhu et al., 2021)	0
Oxygen Saturation *N* = 3	0	3 (Karst et al., 2007; Strong et al., 2016; Dellovo et al., 2019)

### 3.8. Meta-analysis of auricular stimulation on cardiovascular parameters

#### 3.8.1. Blood pressure

**Figure 5:**
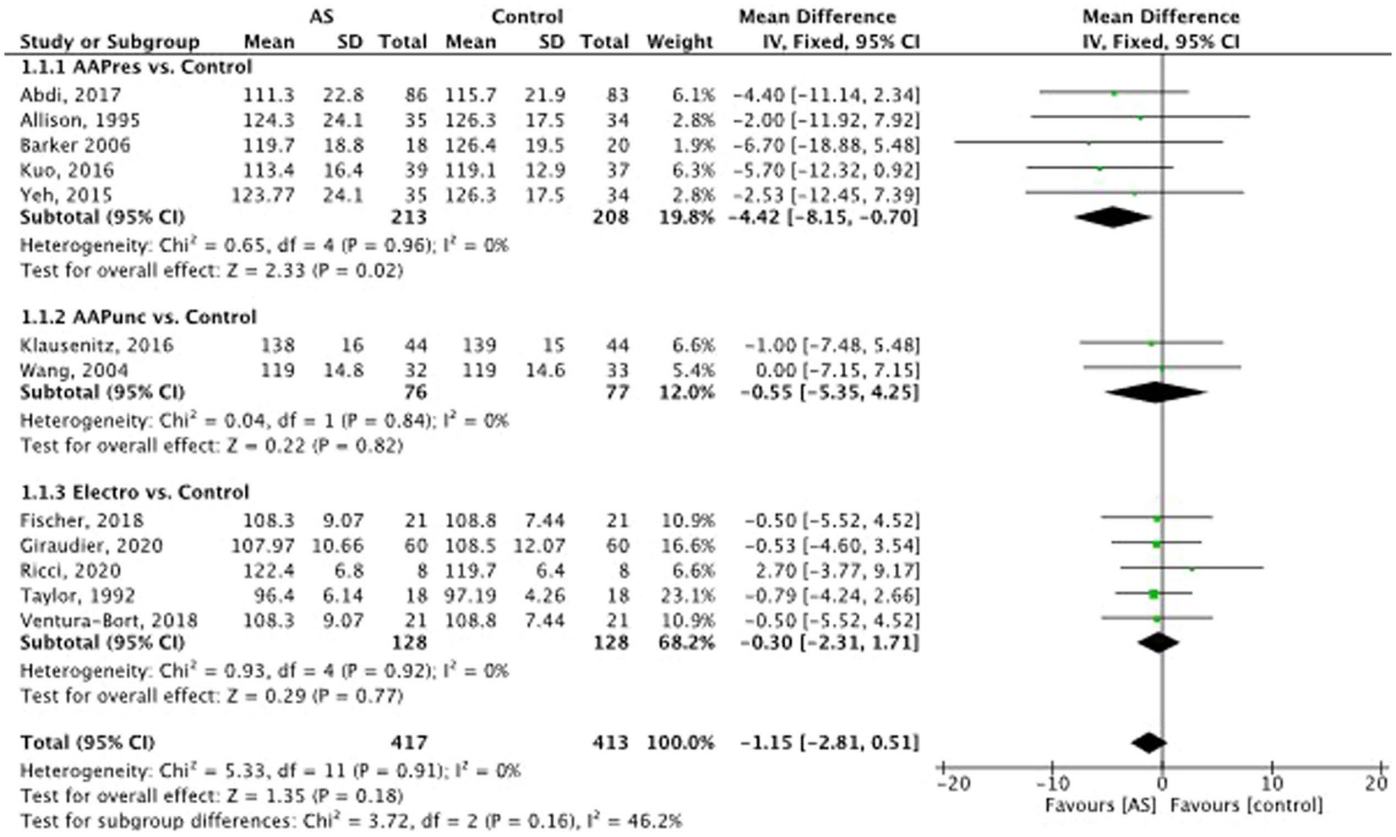
Systolic blood pressure: auricular stimulation vs control.

**Figure 6:**
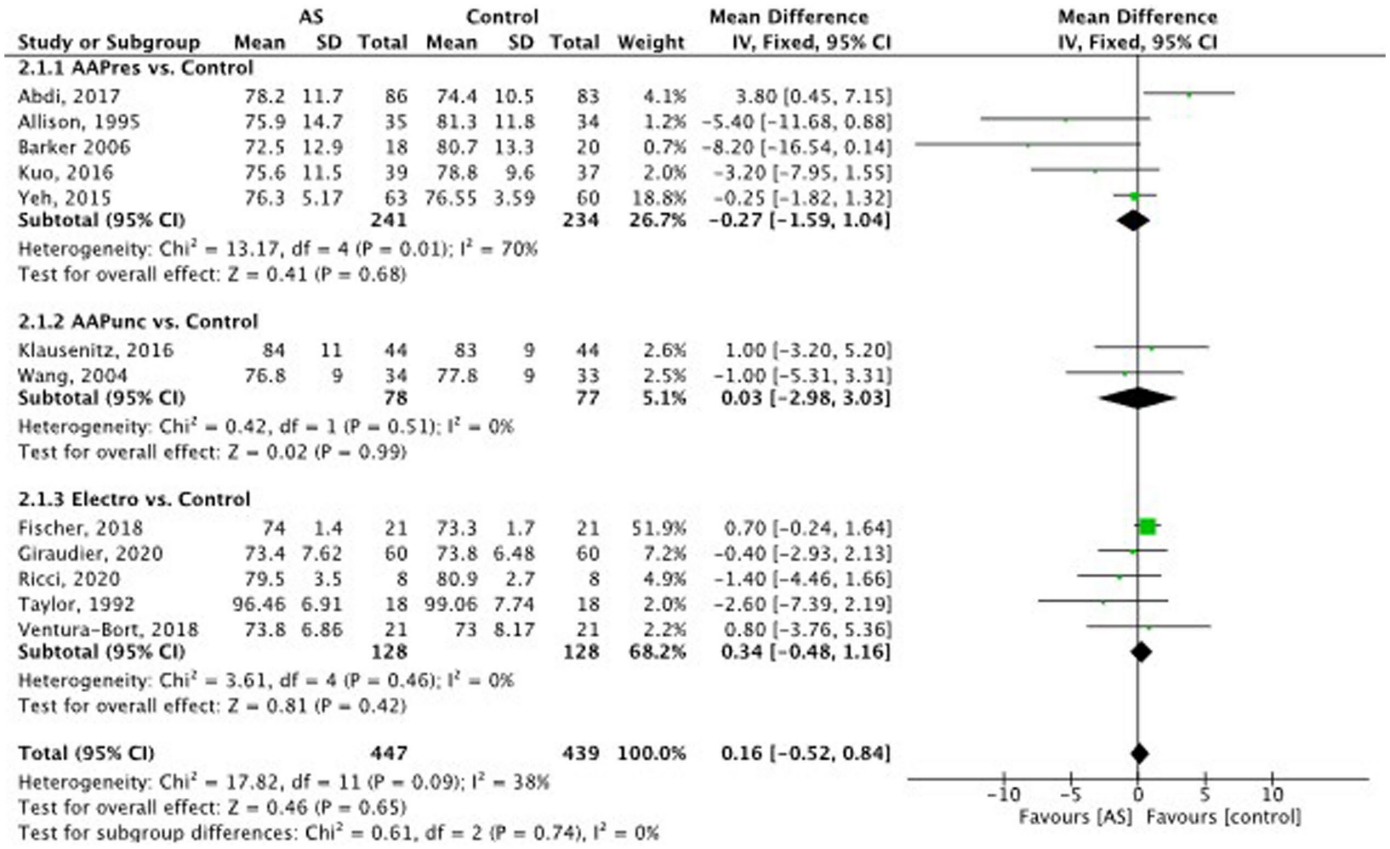
Diastolic blood pressure: auricular stimulation vs control.

Twelve studies could be included for the meta-analysis regarding systolic and diastolic BP (see Figures 5, 6) (Allison et al., 1995; Wang et al., 2004; Yeh et al., 2015; Klausenitz et al., 2016; Kuo et al., 2016; Abdi et al., 2017; Fischer et al., 2018; Ventura-Bort et al., 2018; Giraudier et al., 2020; Ricci et al., 2020). Compared to the control methods, AS did not have a significant influence on systolic BP. MD = −1.15, 95% CI (−2.81 to 0.51), *p* = 0.16.

#### 3.8.2. Heart rate

**Figure 7:**
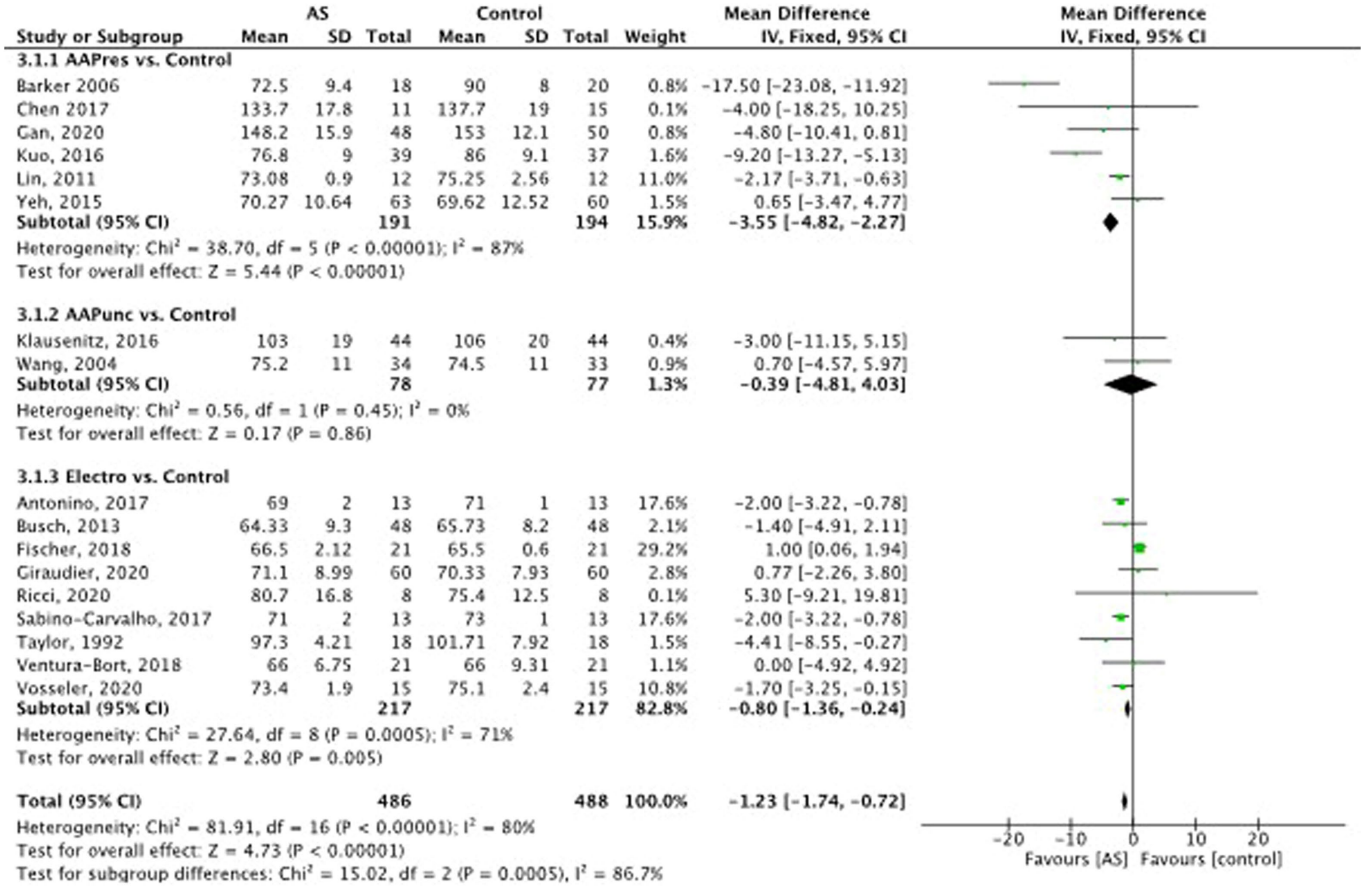
Heart rate: auricular stimulation vs control.

Seventeen studies (Taylor and Lee, 1992; Wang et al., 2004; Barker et al., 2006; Lin et al., 2011; Busch et al., 2013; Yeh et al., 2015; Klausenitz et al., 2016; Kuo et al., 2016; Antonino et al., 2017; Chen et al., 2017; Sabino-Carvalho et al., 2017; Fischer et al., 2018; Ventura-Bort et al., 2018; Villani et al., 2019; Gan et al., 2020; Giraudier et al., 2020; Ricci et al., 2020; Vosseler et al., 2020) had data regarding HR (see Figure 7). The results suggest that auricular stimulation significantly reduces heart rate compared with control procedures MD = −1,23, 95% CI (−1.74 to −0.72), *p* = 0.0005. No drop of HR in any of the trials were regarded as adverse event. The strongest decrease was achieved in the auricular acupressure subgroup. Here Kuo et al. (2016) conducted a trial with 80 postpartum women, with the primary outcome to relieve stress and anxiety. The intervention group received auricular acupressure together with routine care over 4 days compared to a control group that received routine care only. The acupressure was administered by a researcher. HR was measured as secondary outcome parameter and showed to be lower in the intervention group compared to the routine care group by a mean of 9.2 bpm [CI95% 13.27, 5.13]. Barker et al. (2006) conducted a trial in 38 elder patients with fresh hip fracture to reduce pain and anxiety on the way to the hospital. Acupressure was administered by the paramedics before the transfer and compared to sham acupressure. The intervention group had significantly lower pain and anxiety and heart rates arriving at the hospital by a mean of 18 bpm. Interestingly this trial was a double-blind trial since the paramedics did not know if they administered at real or sham points on the auricle.

The reduction of HR in the electrostimulation group was statistically significant, but not clinically relevant- the strongest reduction was achieved in the experiment by Taylor and Lee (1992) on 90 healthy volunteer students, who received in a 5-armed randomized trial design 30 min of TENS electrostimulation of different intensities (0–5 kHz, 0-10 mA) to both earlobes or no stimulation. This safety trial monitored HR and blood pressure, and anxiety. The strongest reductions of HR were achieved in the 100 Hz group resulting in a mean 89.22 bpm + − SD 6.13 vs. 101.71 bpm + − SD 7.92 in the placebo TENS group.

### 3.9. Sensitivity analysis

Although the outlier that was excluded in favor of auricular stimulation in the sensitivity analysis, a significant reduction in heart rate could still be detected: MD (95%CI)-1.09 [−1.61, −0.58], p 0.0001.

### 3.10. Heart rate variability

**Figure 8:**
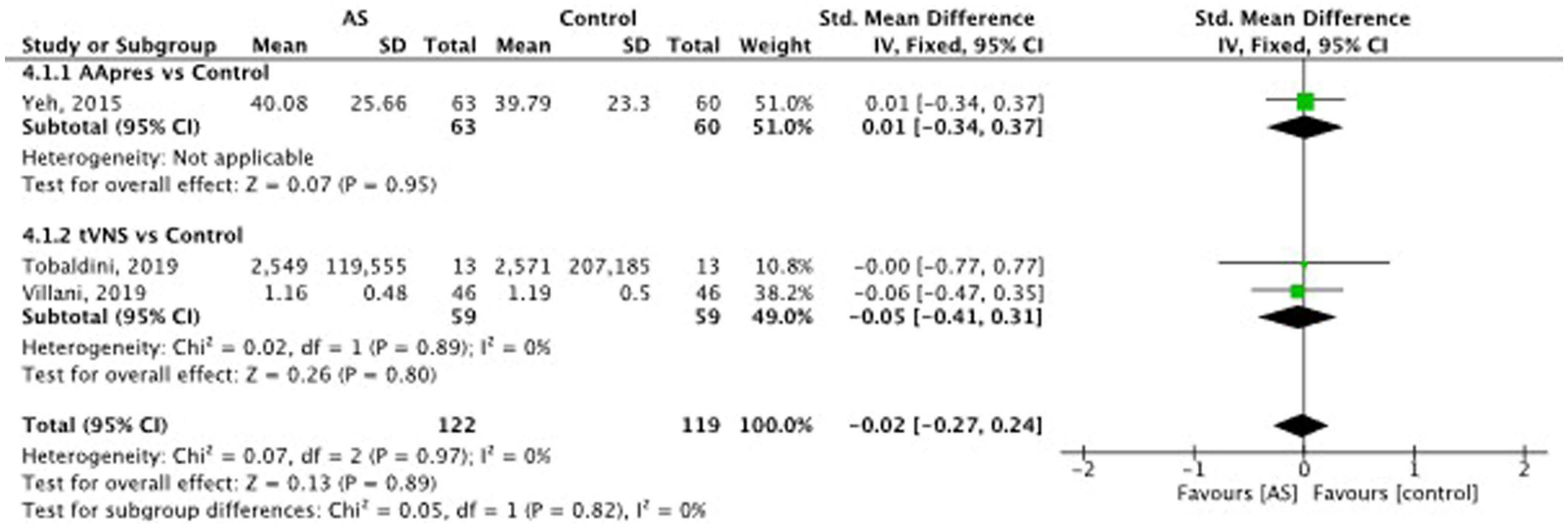
Heart rate variability: auricular stimulation vs control.

**Figure 9:**
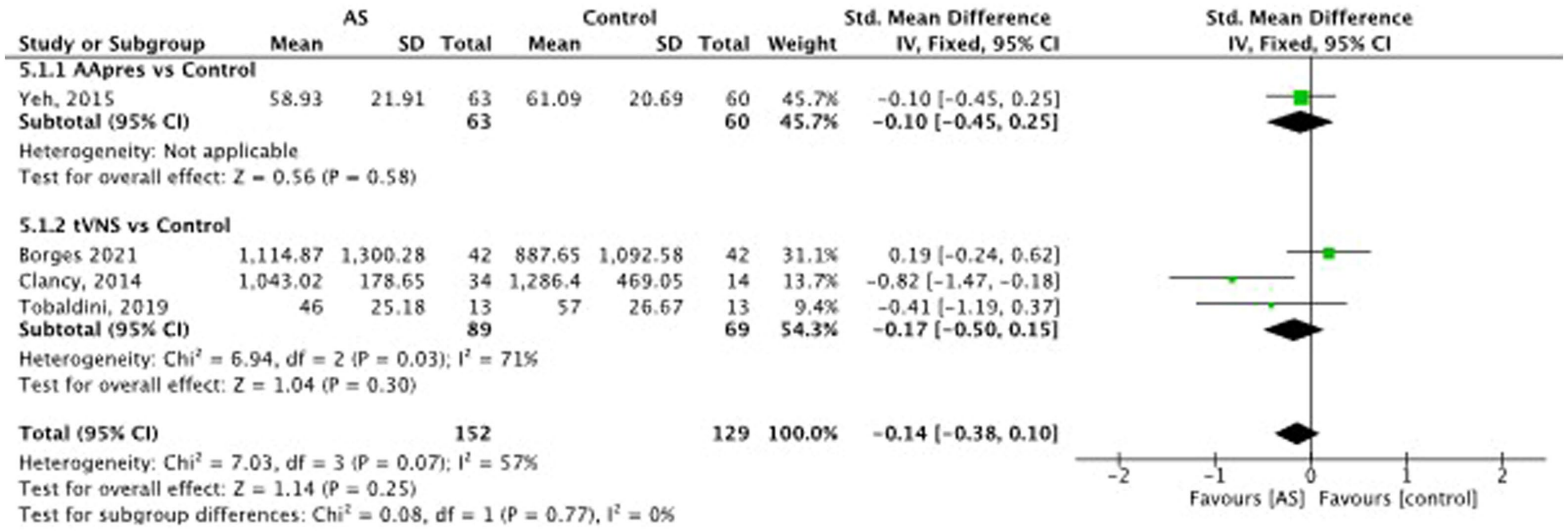
Heart rate variability (high frequency): auricular stimulation vs control.

**Figure 10:**
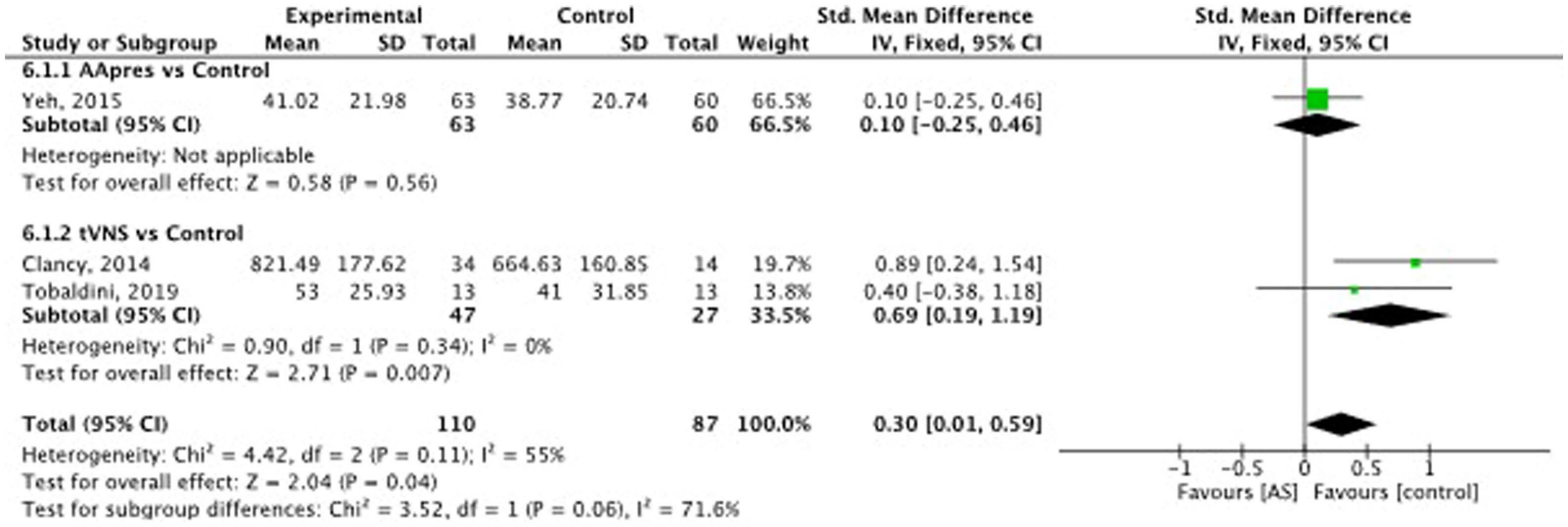
Heart rate variability (low frequency): auricular stimulation vs control.

**Figure 11:**
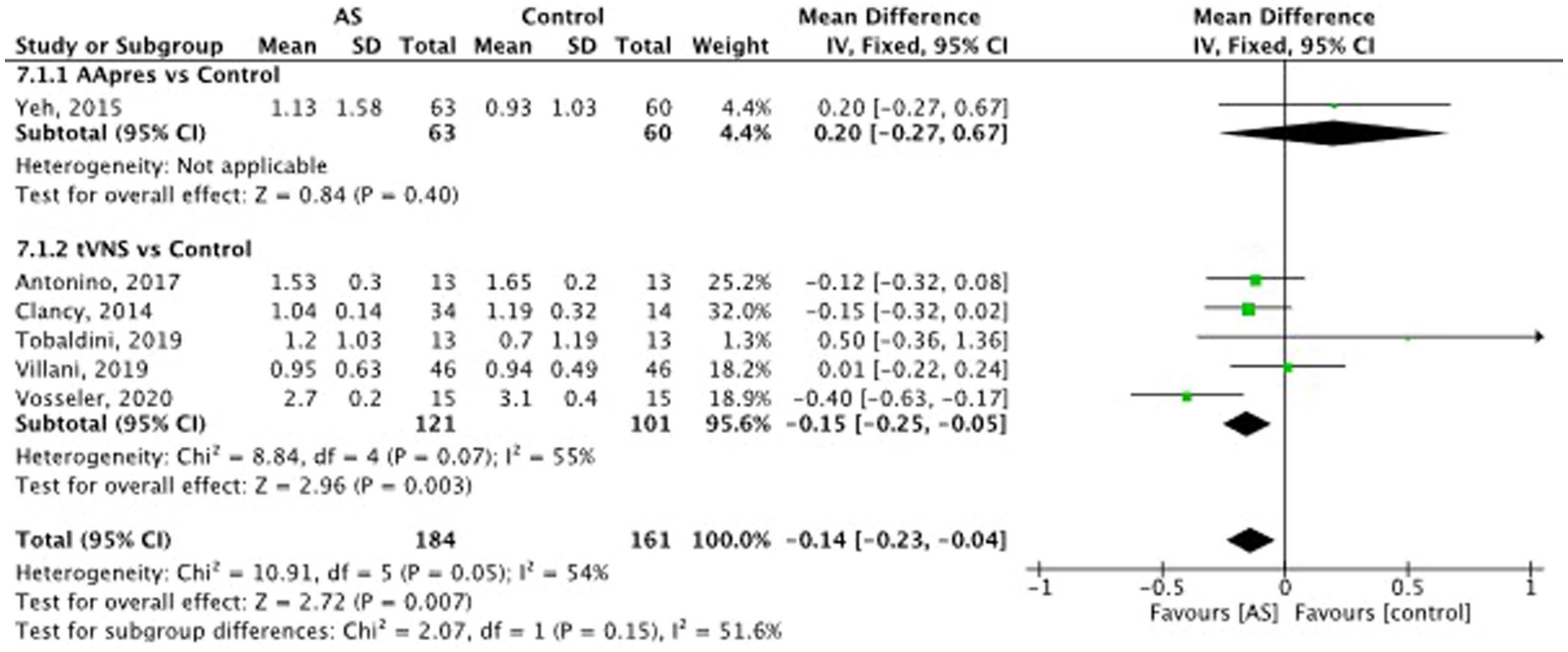
Low-frequency/high-frequency ratio (LF/HF): auricular stimulation vs control.

Auricular stimulation does not significantly influence HRV compared to control procedures in 3 studies SMD = −0.02, 95% CI (−0.27 to 0.24), *p* = 0.82 (see Figure 8). Examining the high frequency (HF) power within HRV, auricular stimulation could not show any significant influence compared to the control procedures SMD = −0.14, 95% CI (−0.38 to 0.10), *p* = 0.25 (see Figure 9). Three studies had data to calculate low frequency (LF) power. Thereby, the experimental group leads to increased LF power. SMD = 0.30, 95% CI (0.01 to 0.59), *p* = 0.04 (see Figure 10). Regarding the LF/HF Ratio the analysis of 6 studies demonstrated, that auricular stimulation had a significant effect over control procedures with lower ratios in the AS group (MD = −0.14, 95% CI (−0.23 to 0.04), *p* = 0.007) (see Figure 11).

In order to measure the effects of the electrical stimulation of the ABVN on the autonomic nervous system, Clancy and her colleagues (2014) (Clancy et al., 2014) conducted an experimental RCT in 48 healthy participants. 34 received 15 min of 30 Hz tVNS with an intensity to the level of sensory threshold on the inner and outer surface of the tragus, compared to a sham group with inactivated electrodes on the tragus. HRV was measured and a significant decrease in LF/HF ratio during active tVNS could be shown in comparison to the sham group that did not show a significant decrease.

Another experimental cross- over RCT on 15 healthy men by Vosseler et al. (2020), measured the effects every 30 min in 120 min of tVNS with a frequency of 25 Hz and 2.5 mA on the cymba conchae, compared to the earlobe in the sham group. The earlobe is supposed to be free of vagal nerve fibers. Endocrine and metabolic parameters and on peripheral vagal activity during an oral glucose tolerance test were the outcome parameters. Significant reduction in LF/HF ratio were achieved at the end of the stimulation (at 120 min).

Antonino et al. (2017) tested the hypothesis that tVNS acutely improves spontaneous cardiac baroreflex sensitivity (cBRS) and autonomic modulation in a cross over RCT in 13 healthy men. Bilateral tVNS on the tragus over 15 min with 30 Hz between 10 and 50 mA up to the level of sensory threshold was compared to sham tVNS with no current and tVNS on the earlobe. HRV was measured during the stimulation and tVNS significantly reduced LF/HF ratio and returned to baseline values during recovery.

### 3.11. Oxygen saturation

**Figure 12:**
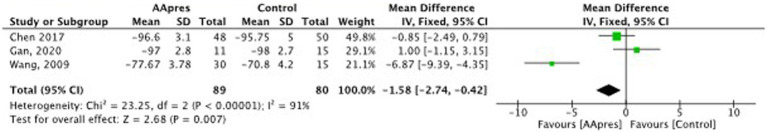
Oxygen saturation: auricular stimulation vs control.

Continuous data of O2 saturation were provided in 3 trials (WANG et al., 2009; Chen et al., 2017; Gan et al., 2020). In 2 trials (Chen et al., 2017; Gan et al., 2020) they were part of secondary parameters to objectify indirectly the intensity of pain (intense crying) in neonates undergoing unpleasant examination procedures. In both trials acupressure led to significantly lower pain perception, though only in the trial by Gan et al. (2020) on 100 neonates undergoing painful eye inspection, AS prevented significantly from intense crying with subsequent O2 reduction. The trial by WANG et al. (2009) was conducted in 45 adults with Obstructive Sleep Apnoe Syndrome (OSAS) and impaired O2 saturation at night-a 3 month lasting acupressure led to relevant improvement of sleeping patterns and a significant improvement of nocturnal O2 levels after the end of treatment (see Figure 12).

### 3.12. Baroreflex sensitivity and skin conductance

AS led to increased baroreflex sensitivity in two studies with tVNS (see Supplementary Figure S2); two studies showed no difference between groups in Skin conductance MD = −0.47, 95% CI (−1.00 to 0.05), *p* = 0.08 (see Supplementary Figure S3).

### 3.13. Funding sources

Of the 76 included studies, 27 research groups did not provide funding information. (Ceccherelli et al., 1981; Taylor and Lee, 1992; Wang and Kain, 2001; Wang et al., 2004, 2009; Usichenko et al., 2005, 2006; Barker et al., 2006; Lin et al., 2011; Wetzel et al., 2011; Lu et al., 2012; Arai et al., 2013; Busch et al., 2013; Hein et al., 2013; Capone et al., 2015, 2017; Jacobs et al., 2015; Yeh et al., 2015; Bauer et al., 2016; Burger et al., 2016, 2018; Strong et al., 2016; Yu et al., 2017; Fischer et al., 2018; Dellovo et al., 2019; Borges et al., 2020, 2021). Four trials were partly sponsored by companies (Johnson et al., 1991; Allison et al., 1995; Hasan et al., 2015; Wagenseil et al., 2018). The majority of the studies were funded either by public funds (Napadow et al., 2012; Yeo et al., 2014; Sellaro et al., 2015; Stavrakis et al., 2015, 2020; Steenbergen et al., 2015; Luo et al., 2016; Abdi et al., 2017; Burger et al., 2017, 2019; Sabino-Carvalho et al., 2017; Badran et al., 2018; Colzato et al., 2018; Ventura-Bort et al., 2018; Keute et al., 2019; Nakahara et al., 2019; Sclocco et al., 2019; Tobaldini et al., 2019; Villani et al., 2019; Giraudier et al., 2020; Wu et al., 2020; Zhu et al., 2021) or were investigator initiated (Karst et al., 2007; Usichenko et al., 2007, 2020; la Marca et al., 2010; Black et al., 2011; Taylor et al., 2013; Clancy et al., 2014; Laqua et al., 2014; Klausenitz et al., 2016; Kuo et al., 2016; Antonino et al., 2017; Chen et al., 2017; de Couck et al., 2017; Borges et al., 2019; Bretherton et al., 2019; Gan et al., 2020; Gauthey et al., 2020; Hendawy and Abuelnaga, 2020; Ricci et al., 2020; Staley et al., 2020; Koenig et al., 2021).

### 3.14. Safety of intervention

Out of the 76 included studies, 37 studies reported adverse events. Overall, no serious side effect occurred in any study. 21 studies reported minor side effects (Allison et al., 1995; Usichenko et al., 2005, 2006, 2007; la Marca et al., 2010; Lu et al., 2012; Laqua et al., 2014; Hasan et al., 2015; Jacobs et al., 2015; Stavrakis et al., 2015; Bauer et al., 2016; Abdi et al., 2017; Burger et al., 2018, 2019; Fischer et al., 2018; Ventura-Bort et al., 2018; Villani et al., 2019; Gan et al., 2020; Giraudier et al., 2020; Ricci et al., 2020; Wu et al., 2020), the most common side effects are: local pain at ear stimulation side (8 trials), erythema (6 trials), headache (5 trials) and skin irritation (5 trials).

### 3.15. GRADE assessment

The overall GRADE assessment for the outcomes systolic and diastolic blood pressure, heart rate, heart rate variability was performed using the GRADE tool. Recommendations of AS for reduction of heart rate were low and very low in the other outcomes (see Supplementary table S1).

## 4. Discussion

In the present systematic review, we have screened various parameters as potential biomarkers for the effects of auricular stimulation on cardiovascular function of the human body. Although these parameters were not the primary outcomes in the RCTs included in this review, we have found a clinically significant reduction of HR as well as reduced LF/HF ratio after auricular stimulation compared to control procedures. This finding sounds physiologically plausible, since the main mechanism of auricular stimulation suggested is the modulation of autonomic nervous system (Usichenko et al., 2017) and both effects found (reduction of HF and LF/HF ratio) are consistent with the physiological reaction to the stimulation of the auricular branch of the vagal nerve (Peuker and Filler, 2002).

No other cardiovascular parameters (blood pressure, oxygen saturation, baroreflex sensitivity) were changed significantly.

The electrical stimulation of the parasympathetic nervous system via the auricular branch of the vagus nerve (Alderman’s nerve or Arnold’s nerve) (Peuker and Filler, 2002), raised concerns about cardiovascular safety of AS, especially in an elderly population with cardiovascular comorbidities. These concerns are based on experimental investigations with direct stimulation of cervical vagal nerves in dogs that demonstrated the more pronounced effect of right vagal nerve stimulation on bradycardia in comparison with the left side stimulation (Ardell and Randall, 1986). However, the simple theoretical transfer of this effect in case of AS is not justified, since the neurocircuitry of transcutaneous vagal nerve stimulation differs from the direct form, where the left-sided AS is suggested as equally safe as the right-sided AS (Chen et al., 2015). Moreover, it is well known, that especially in patients with heart diseases the sympathetic part of autonomous nervous system is pathologically activated and is the target for various pharmacological interventions (e.g., block of beta-adrenergic receptors) (de Lucia et al., 2019). Thus, in this cohort of patients, AS can serve as an additional non-pharmacologic method of myocardial protection due to reduction of heart rate and favorable modulation of the tone of autonomic nervous system. Indeed, tVNS is being investigated for modulating arrhythmias such as in atrial fibrillation (Yu et al., 2013; Stavrakis et al., 2015). It was demonstrated that tVNS relieved angina pectoris complaints, reduced heart rate and blood pressure and reduced the incidence of heart failure in comparison with the control group due to an inhibition of norepinephrine release from sympathetic nerves with subsequent dilation of cardiac microcirculatory vessels and improved left ventricular contractility in patients with severe coronary artery disease (Zamotrinsky et al., 2001).

One of the strengths of our work is that it has summarized the effects on cardiovascular factors of a broad range of AS, including traditional ways such as ear-acupuncture and modern ways such as electrical tVNS.

One explanation for the comparably larger effects in the acupressure groups (sysBP, HR) compared to the electrical stimulation such as in tVNS are the much longer duration of the stimulation in the acupressure group; here small beads or plant seeds are stuck with a tape to the auricle and remain *in situ* over several weeks, while the experimental trials with tVNS often apply electrical stimulation only maximum over several hours.The discrepancy of the results of the meta-analysis and the descriptive analysis of non-continuous data especially in the HR could be explained with the fact, that HR was used as a safety parameter in a large number of trials. Relevant changes were only reported if they were considered a safety issue, data of smaller changes were not reported.

### 4.1. Limitations

First of all the analysis was conducted with secondary outcome parameters, so conclusions are limited. Some of the trials with the strongest reduction in HR for example were investigating the effect of acupressure on anxiety and stress and pain as primary outcomes, therefore the cardiovascular effects could have been a secondary effect to the relief of anxiety and/ or pain (Barker et al., 2006; Kuo et al., 2016). Next, the literature search was limited to English and other European languages. A relevant number of studies is published in Chinese and could therefore not be evaluated. Furthermore, the meta-analysis offers limited power due to the moderate study quality of the included studies. In some subgroups the numbers of included studies of included studies are so small, that conclusions about effects cannot be drawn.

## 5. Conclusion

The findings of this systematic review support previously suggested mechanism of AS via activation of parasympathetic nervous system. The reduction of heart rate was clinically safe, the potential myocardial protective effect due to avoiding of tachycardias and modulating of autonomic imbalance should be clarified in future original investigations.

## Data availability statement

The original contributions presented in the study are included in the article/Supplementary material, further inquiries can be directed to the corresponding authors.

## Author contributions

JD and KH: data collection. KH and MC: meta-analysis. All authors: drafting of the manuscript.

## Funding

KH received a research grant no. 02042022 from the International Society of Chinese Medicine, Societas Medicinae Sinensis, Munich, Germany. The society had no role in the development of the protocol.

## Conflict of interest

The authors declare that the research was conducted in the absence of any commercial or financial relationships that could be construed as a potential conflict of interest.

## Publisher’s note

All claims expressed in this article are solely those of the authors and do not necessarily represent those of their affiliated organizations, or those of the publisher, the editors and the reviewers. Any product that may be evaluated in this article, or claim that may be made by its manufacturer, is not guaranteed or endorsed by the publisher.

## Supplementary material

The Supplementary material for this article can be found online at: https://www.frontiersin.org/articles/10.3389/fnins.2023.1227858/full#supplementary-material

## Footnotes

^1^Available at www.covidence.org
