# Supplementary material for: Cardiovascular effects of auricular stimulation -a systematic review and meta-analysis of randomized controlled clinical trials
Source: Front Neurosci. 2023 Sep 1;17:1227858. doi: 10.3389/fnins.2023.1227858 (PMC10505819; doi:10.3389/fnins.2023.1227858)
Supplement: Supplementary file 1 [file Data_Sheet_1.docx]

Supplementary Material

Cardiovascular effects of auricular stimulation -a systematic review and meta-analysis of randomized controlled clinical trials

**Authors:** Kevin Hua^1^, MD, Mike Cummings^2^, MD, Miriam Bernatik^3^, MD, Benno Brinkhaus^1^, MD, PhD; Taras Usichenko^4,5^, MD, PhD, Joanna Dietzel^1^, MD

**Correspondence:** Joanna Dietzel, Institute for Social Medicine, Epidemiology and Health Economics, Charité University Medicine, Luisenstr. 57, 10117 Berlin, Germany; [Joanna.Dietzel@charite.de](mailto:Joanna.Dietzel@charite.de)

# Supplementary Tables

**Table S1 GRADE Evaluation**

**Question:** Auricular stimulation compared to control for cardiovascular biomarker

**Bibliography:** . Auricular Stimualtion for Biomarker (Cardiovascular).

| **Certainty assessment** | | | | | | | **№ of patients** | | **Effect** | | **Certainty** |
| --- | --- | --- | --- | --- | --- | --- | --- | --- | --- | --- | --- |
| **№ of studies** | **Study design** | **Risk of bias** | **Inconsistency** | **Indirectness** | **Imprecision** | **Other considerations** | **auricular stimulation** | **control** | **Relative (95% CI)** | **Absolute (95% CI)** |  |
| 12 | randomised trials | serious^a^ | not serious | not serious | very serious^b^ | none | 417 | 413 | - | MD 1.15 lower (2.81 lower to 0.51 higher) | ⨁◯◯◯ Very low |
| 12 | randomised trials | serious^,a^ | not serious | not serious | very serious^b^ | none | 447 | 439 | - | MD 0.16 higher (0.52 lower to 0.84 higher) | ⨁◯◯◯ Very low |
| 17 | randomised trials | very serious^d,e^ | serious^f^ | not serious | not serious | none | 486 | 488 | - | MD 1.23 lower (1.74 lower to 0.72 lower) | ⨁◯◯◯ Very low |
| 16 | randomised trials | very serious^d,e^ | not serious | not serious | not serious | None | 468 | 468 | - | MD 1.09 lower (1.61 lower to 0.58 lower) | ⨁⨁◯◯ Low |
| 3 | randomised trials | serious^d^ | not serious | not serious | serious^g^ | none | 122 | 119 | - | SMD 0.02 lower (0.27 lower to 0.24 higher) | ⨁⨁◯◯ Low |
| 4 | randomised trials | serious^a^ | not serious | not serious | serious^g^ | none | 152 | 129 | - | SMD 0.14 lower (0.38 lower to 0.1 higher) | ⨁⨁◯◯ Low |
| 3 | randomised trials | serious^d^ | not serious | not serious | serious^g^ | none | 110 | 87 | - | SMD 0.3 higher (0.01 higher to 0.59 higher) | ⨁⨁◯◯ Low |
| 6 | randomised trials | serious^a^ | serious^h^ | not serious | serious^b^ | none | 184 | 161 | - | MD 0.14 lower (0.23 lower to 0.04 lower) | ⨁◯◯◯ Very low |
| 2 | randomised trials | serious^i^ | serious^j^ | not serious | not serious | none | 27 | 27 | - | MD 1.58 higher (1.17 higher to 1.98 higher) | ⨁⨁◯◯ Low |
| 2 | randomised trials | serious^i^ | not serious | not serious | serious^g^ | none | 63 | 63 | - | MD 0.47 lower (1 lower to 0.05 higher) | ⨁⨁◯◯ Low |
| 3 | randomised trials | not serious | serious^k^ | not serious | serious^m^ | none | 89 | 80 | - | MD 1.57 higher (0.41 higher to 2.72 higher) | ⨁⨁◯◯ Low |

**CI:** confidence interval; **MD:** mean difference

**Explanations**

a. Three studies have shown increased risk of bias. (maximum high risk of bias on "blinding personal")

b. The higher confidence intervall breaches our SMD 0.2 taken as clinically important differences MCID vs control, but also crosses the line of no effect.

c. Three studies provided 3 items with unsure risk of bias.

d. Two studies breaches a high risk of bias. (maximum high risk of bias on "blinding personal")

e. Eight studies have 3 items which contains unclear risk of bias

f. High heterogenity is detected in overal calculation (I2=80%)

g. Results are breaching the line of no effect

h. High heterogenity detected among included studies (I2=54%).

i. One study reaches a overall high risk of bias (maximum high risk of bias on "blinding personal")

j. Included studies have a high heterogenity (I2=98%)

k. High heterogenity among included studies (I2= 91%).

m. The lower confidence intervall braches our SMD 0,5 taken as clinically important differences (MCID) vs. control

**Table S2. Overview of stimulation parameters on anatomic regions of pinna and research question. ABVN: auricular branch of the vagus nerve; GAN: great auricular nerve; ATN: auriculo- temporal nerve; nr: not reported, ne: no electro-stimulation.**

| First author, year | innervation | auricular region | current intensity [mA] | pulse width [µS] | frequency [Hz] | Main research question |
| --- | --- | --- | --- | --- | --- | --- |
| Lamarca 2010 [86] | ABVN | cavum conchae inferior | nr | 550 | 10 | Respiratory sinus arrhythmia |
| Sclocco 2019 [23] | ABVN | cymba conchae | 1,7 | 450 | 25 | Vagal activity |
| Gauthey 2020 [77] | ABVN | cymba conchae | 3,5 | 200 | 20 | Effect on sympathicus |
| Wu 2020 [27] | ABVN | cymba conchae | nr | 300 | 20 | Subacute ischemic stroke patients |
| Hein 2013 [30] | ABVN | cavity of conchae | 0,6 | nr | 1,5 | Depression |
| Borges 2021 [81] | ABVN | cymba conchae | 0,94 | 250 | 25 | Neurophysiological and cognitive effects |
| Tobaldini 2019 [44] | ABVN | cymba conchae | 1-1 | 20000 | 25 | Orthostatic stress |
| Giraudier 2020 [45] | ABVN | cymba conchae | 2 | 250 | 25 | Word recognition memory |
| Burger 2017 [46] | ABVN | cymba conchae | 0,5 | 250 | 25 | Neurocognitive extinction training |
| Keute 2019 [48] | ABVN | cymba conchae | 3 | 200 | 25 | Visual bistable perception |
| Burger 2016 [49] | ABVN | cymba conchae | 0,5 | 250 | 25 | Fear extinction |
| Koenig 2019 [50] | ABVN | cymba conchae | 0,5 | 250 | 1 | Major depressive disorder |
| Hasan 2015 [51] | ABVN | cymba conchae | nr | 250 | 25 | Stable schizophrenia |
| Borges 2019 [56] | ABVN | cymba conchae | 1 | 250 | 25 | Heart rate variability |
| Burger 2018 [57] | ABVN | cymba conchae | 0,5 | 250 | 25 | Fear extinction |
| Zhu 2021 [58] | ABVN | cymba conchae | 1 | 500000 | 25 | Functional dyspepsia |
| Laqua 2014 [60] | ABVN | concha and mastoid | nr | 200 | 51 | Pain threshold |
| Sellaro 2015 [61] | ABVN | cavity of conchae | 0,5 | 250 | 25 | Neural activity |
| Vosseler 2020 [63] | ABVN | cymba conchae | 2,5 | nr | 25 | Oral glucose tolerance test |
| Steenbergen 2015 [66] | ABVN | cymba conchae | 0,5 | 250 | 25 | Neurocognitive tasks |
| Fischer 2018 [67] | ABVN | cymba conchae | 1.1 | 250 | 25 | Arousal |
| Burger 2019 [69] | ABVN | cymba conchae | 0,5 | 250 | 25 | Reduction of negative thought intrusions |
| Ventura bort 2018 [70] | ABVN | cymba conchae | 1.1 | 250 | 25 | Locus coeruleus-norepinephrine system |
| Borges 2020 [71] | ABVN | cymba conchae | 2,19 | 250 | 25 | Core executive functions |
| Staley 2020 [31] | ABVN | cymba conchae | nr | 300 | 25 | Hypertension |
| Busch 2013 [43] | ABVN/ATN | tragus | 1.1 | 250 | 25 | Pain processing |
| Hendawy 2020 [21] | ABVN/GAN | fossa triangularis, antitragus, lobule of auricle, | nr | nr | 2 | Postoperative analgesia in abdominal hysterectomy |
| Napadow 2012 [22] | ABVN/GAN | cymba conchae, antihelix | 0,43 | 450 | 30 | Pain analgesia |
| Decouck 2017 [41] | ABVN/GAN | cymba conchae, cavity of conchae | 0,1 | 250 | 25 | Heart rate variability |
| Johnson 1991 [87] | ABVN/GAN | cavity of conchae | nr | nr | 100 | Pain threshold and autonomic functions |
| Bauer 2016 [42] | ABVN/GAN | cymba conchae | 0,5 | 250 | 25 | Drug resistant epilepsy |
| Antonino 2017 [82] | ABVN/GAN | tragus | 45 | 200 | 30 | Cardiac baroreflex sensitivity and autonomic modulation |
| Badran 2018 [52] | ABVN/GAN | tragus | nr | 500 | 10 | Heart rate effects |
| Colzato 2018 [53] | ABVN/GAN | cymba conchae, cavity of conchae | 0,5 | 250 | 25 | Vagus nerve and creativity |
| Villani 2019 [62] | ABVN/GAN | external ear canal | nr | 250 | 25 | Cardiac interoceptive accuracy |
| Jacobs 2015 [65] | ABVN/GAN | tragus | 5 | 200 | 8 | Associative memory performance |
| Capone 2017 [28] | ABVN/GAN/ATN | tragus | nr | 300 | 20 | Upper limb functionality in chronic stroke |
| Yu 2017 [29] | ABVN/GAN/ATN | tragus | nr | 1000 | 20 | Myocardial ischemia-reperfusion injury |
| Capone 2015 [47] | ABVN/GAN/ATN | tragus | 8 | 300 | 20 | Cerebral cortex excitability |
| Bretherton 2019 [54] | ABVN/GAN/ATN | tragus | 3-1 | 200 | 30 | Sleep, mood and autonomic function |
| Sabino-Carvalho 2017 [83] | ABVN/GAN/ATN | tragus | 45 | 200 | 30 | Spontaneous cardiac baroreflex sensitivity |
| Clancy 2014 [59] | ABVN/GAN/ATN | cavity of conchae, cymba, tragus | 30 | 200 | 30 | Autonomic function |
| Stavrakis 2015 [64] | ABVN/GAN/ATN | tragus | nr | 1000 | 20 | Antiarrhythmic and anti-inflammatory effects |
| Ricci 2020 [68] | ABVN/GAN/ATN | tragus | 6,8 | 500 | 30 | Cerebral cortex activity |
| Stavrakis 2020 [72] | ABVN/GAN/ATN | tragus | 16,8 | 200 | 20 | Paroxysmal atrial flutter |
| Taylor 1992 [13] | GAN | lobule of auricle | 0,3 | nr | 5 | Blood pressure and heart rate, anxiety |
| Wagenseil 2018 [40] | GAN | lobule of auricle | 0,1 | nr | 0,5 | Sleep efficiency |
| Taylor 2013 [80] | GAN | lobule of auricle | 0.1 | nr | 0,5 | pain control in fibromyalgia |
| Allison 1995 [16] | ABVN/GAN | crus of helix, concha, cymba | ne | ne | ne | Weight reduction |
| Lu 2012 [17] | ABVN/GAN | antitragus, cymba conchae, cavity of conchae, fossa triangularis | ne | ne | ne | Psoriasis |
| Yeh 2015 [18] | ABVN/GAN | cymba conchae, cavity of conchae, fossa triangularis, crus inferior, antitragus | ne | ne | ne | Hypertension |
| Usichenko 2005 [19] | ABVN/GAN | crura inferior of anthelix, fossa triangularis, antitragus, cavity of conchae | ne | ne | ne | Complementary analgesia after total hip arthroplasty |
| Usichenko 2007 [20] | ABVN/GAN | fossa triangularis, cavity of conchae | ne | ne | ne | Ambulatory knee surgery |
| Wetzel 2011 [24] | ABVN/GAN | fossa triangularis, cavity of conchae, anthelix | ne | ne | ne | Intraoperative analgesic requirement during total hip arthroplasty |
| Usichenko 2006 [25] | ABVN/GAN | cavity of conchae, lobule of auricle, fossa triangularis, crura inferior of anthelix | ne | ne | ne | Analgesic requirements during total hip arthroplasty |
| Kuo 2016 [55] | ABVN/GAN | fossa triangularis | ne | ne | ne | Postpartum improvement of autonomic parameter |
| Wang 2001 [76] | ABVN/GAN | fossa triangularis | ne | ne | ne | Anxiety |
| Gan 2020 [32] | ABVN/GAN | fossa triangularis, lobule of auricle, tail of helix, | ne | ne | ne | Pain during preterm infants examination |
| Kovacic 2020 [34] | ABVN/GAN | lobule of auricle, fossa triangularis | ne | ne | ne | Pediatric functional abdominal pain disorders |
| Luo 2016 [35] | ABVN/GAN | fossa triangularis | ne | ne | ne | Preoperative anxiety |
| Barker 2006 [36] | ABVN/GAN | fossa triangularis, tragus, crura inferior of anthelix | ne | ne | ne | Preoperative anxiety |
| Black 2011 [78] | ABVN/GAN | crus inferior of anthelix, cymba conchae, cavity of conchae, fossa triangularis | ne | ne | ne | Anxiety |
| Nakahara 2019 [89] | ABVN/GAN | cavity of conchae | ne | ne | ne | Heart and vascular response |
| Arai 2013 [39] | ABVN/GAN | crus of helix, fossa triangularis | ne | ne | ne | Postoperative heart rate variability |
| Yeo 2014 [84] | ABVN/GAN | tragus, cymba conchae, cavity of conchae, fossa triangularis | ne | ne | ne | Obesity |
| Strong 2016 [15] | ABVN/GAN | cavity of conchae | ne | ne | ne | Dyspnea intensity in lung cancer patients |
| Wang 2009 [73] | ABVN/GAN | cymba conchae, cavity of conchae, lobule of auricle, fossa triangularis, anthelix, crura inferior of anthelix | ne | ne | ne | Obstructive sleep apnea syndrome |
| Karst 2007 [88] | ABVN/GAN | lobule of auricle, fossa triangularis, antitragus | ne | ne | ne | Dental anxiety |
| Szechenyi 2015 [85] | ABVN/GAN | cymba conchae, cavity of conchae, crura inferior of anthelix, fossa triangularis | ne | ne | ne | Stress reduction |
| Killeen 2002 [74] | ABVN/GAN | crura inferior of anthelix, cymba conchae, cavity of conchae, fossa triangularis | ne | ne | ne | Cocaine craving |
| Wang 2004 [26] | ABVN/GAN/ATN | lobule of auricle, fossa triangularis, tragus | ne | ne | ne | Parental preoperative anxiety |
| Usichenko 2020 [75] | ABVN/GAN/ATN | antitragus, tragus, cymba conchae, cavity of conchae, fossa triangularis | ne | ne | ne | Exam anxiety |
| Abdi 2017 [12] | ABVN/GAN/ATN | tragus, lobule of auricle, crus of helix, fossa triangularis, cymba conchae | ne | ne | ne | Hypertension |
| Chen 2017 [33] | ABVN/GAN/ATN | spine of helix, fossa triangularis, cavity of conchae, tragus, antitragus | ne | ne | ne | Infant pain during heel pricks |
| Lin 2011 [37] | ABVN/GAN/ATN | antitragus, tragus, cymba conchae, cavity of conchae, fossa triangularis | ne | ne | ne | Athletes’ recovery after exercise |
| Ceccherelli 1981 [38] | ABVN/GAN/ATN | fossa triangularis, spine of helix | ne | ne | ne | Reduction of hallucinations after ketamine use |
| Klausenitz 2016 [79] | ABVN/GAN/ATN | tragus, cymba conchae, cavity of conchae, antitragus, fossa triangularis | ne | ne | ne | Exam anxiety |
| Dellovo 2019 [14] | ABVN/GAN/ATN | crus inferior of anthelix, antitragus, cymba conchae, cavity of conchae, lobule of auricle, fossa triangularis | ne | ne | ne | Anxiety |

## Supplementary Figures

See JPEG supp fig. 1, supp fig. 2., supp fig. 3.
