# Supplementary figures and images for: Cardiovascular effects of auricular stimulation -a systematic review and meta-analysis of randomized controlled clinical trials
Source: Front Neurosci. 2023 Sep 1;17:1227858. doi: 10.3389/fnins.2023.1227858 (PMC10505819; doi:10.3389/fnins.2023.1227858)

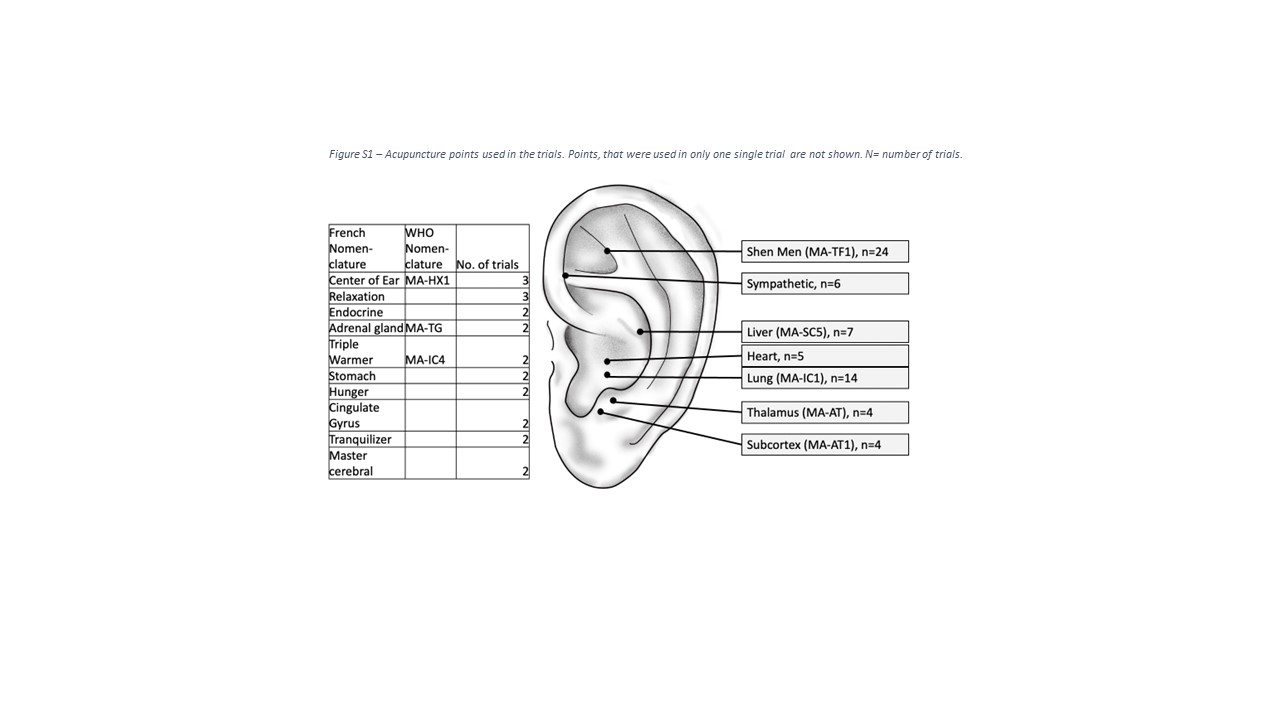

Supplement: Supplementary file 2 [file Image_1.JPEG]

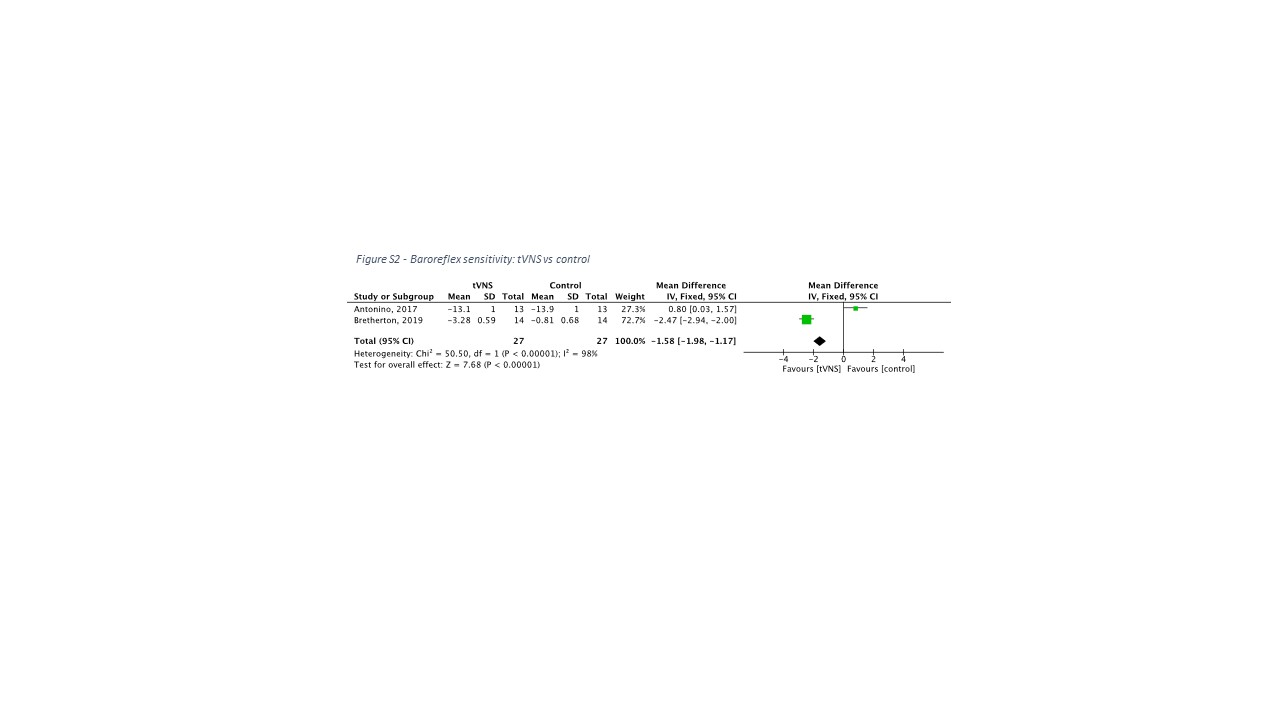

Supplement: Supplementary file 3 [file Image_2.JPEG]

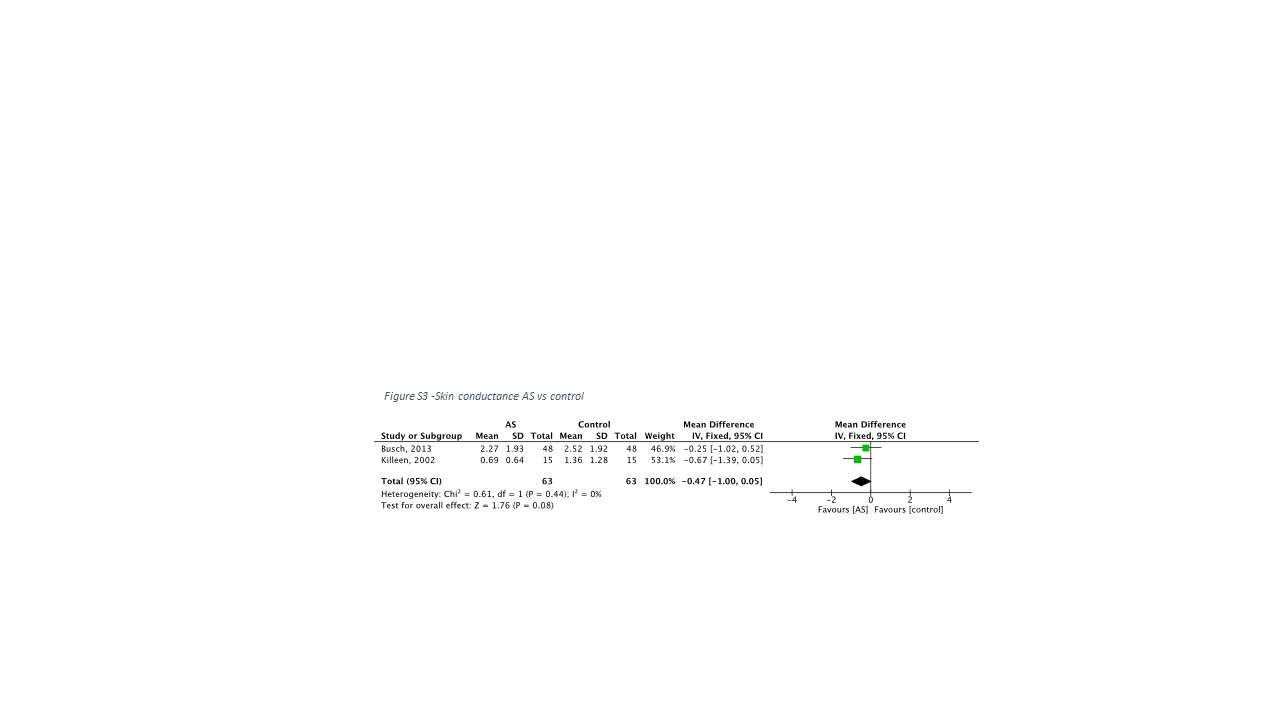

Supplement: Supplementary file 4 [file Image_3.JPEG]
